# Distinct Regulation of Early Trafficking of the NMDA Receptors by the Ligand-Binding Domains of the GluN1 and GluN2A Subunits

**DOI:** 10.1523/JNEUROSCI.0226-24.2025

**Published:** 2025-05-27

**Authors:** Jakub Netolicky, Petra Zahumenska, Anna Misiachna, Marharyta Kolcheva, Kristyna Rehakova, Katarina Hemelikova, Stepan Kortus, Emily Langore, Jovana Doderovic, Marek Ladislav, Jan Korabecny, Michal Otyepka, Martin Srejber, Martin Horak

**Affiliations:** ^1^Department of Neurochemistry, Institute of Experimental Medicine of the Czech Academy of Sciences, Prague 14220, Czech Republic; ^2^Department of Physiology, Faculty of Science, Charles University in Prague, Prague 12843, Czech Republic; ^3^Biomedical Research Center, University Hospital Hradec Kralove, Sokolska 581, Hradec Kralove 500 05, Czech Republic; ^4^IT4Innovations, VSB – Technical University of Ostrava, Ostrava-Poruba 708 00, Czech Republic; ^5^Regional Center of Advanced Technologies and Materials, Czech Advanced Technology and Research Institute (CATRIN), Palacky University Olomouc, Olomouc 779 00, Czech Republic

**Keywords:** endoplasmic reticulum, glutamate receptor, Golgi apparatus, hippocampal neuron, ion channel, pathogenic variant

## Abstract

*N*-Methyl-d-aspartate receptors (NMDARs) play a crucial role in excitatory neurotransmission, with numerous pathogenic variants identified in the GluN subunits, including their ligand-binding domains (LBDs). The prevailing hypothesis postulates that the endoplasmic reticulum (ER) quality control machinery verifies the agonist occupancy of NMDARs, but this was tested in a limited number of studies. Using microscopy and electrophysiology in the human embryonic kidney 293 (HEK293) cells, we found that surface expression of GluN1/GluN2A receptors containing a set of alanine substitutions within the LBDs correlated with the measured EC_50_ values for glycine (GluN1 subunit mutations) while not correlating with the measured EC_50_ values for l-glutamate (GluN2A subunit mutations). The mutant cycle of GluN1-S688 residue, including the pathogenic GluN1-S688Y and GluN1-S688P variants, showed a correlation between relative surface expression of the GluN1/GluN2A receptors and the measured EC_50_ values for glycine, as well as with the calculated Δ*G*_binding_ values for glycine obtained from molecular dynamics simulations. In contrast, the mutant cycle of GluN2A-S511 residue did not show any correlation between the relative surface expression of the GluN1/GluN2A receptors and the measured EC_50_ values for l-glutamate or calculated Δ*G*_binding_ values for l-glutamate. Coexpression of both mutated GluN1 and GluN2A subunits led to additive or synergistic alterations in the surface number of GluN1/GluN2A receptors. The synchronized ER release by ARIAD technology confirmed the altered early trafficking of GluN1/GluN2A receptors containing the mutated LBDs. The microscopical analysis from embryonal rat hippocampal neurons (both sexes) corroborated our conclusions from the HEK293 cells.

## Significance Statement

We examined >80 mutant GluN1/GluN2 receptor combinations, including pathogenic and potentially pathogenic variants in the ligand-binding domains (LBDs) of GluN1 and GluN2A subunits. The combination of the experimentally measured (relative surface expression, EC_50_ values) and calculated (Δ*G*_binding_ values, root mean square deviation, and solvent-accessible surface area) parameters revealed that the LBDs of the GluN1 and GluN2A subunits distinctly regulate the early trafficking of GluN1/GluN2A receptors. In addition, we validated a novel system of synchronized release of GluN1/GluN2A receptors from the endoplasmic reticulum. Our findings support the urgency of further detailed research on the regulation of early trafficking of *N*-methyl-d-aspartate receptors, as it may open the avenue to targeted intervention of central nervous system disorders associated with pathogenic variants in GluN subunits.

## Introduction

**Figure 1.**
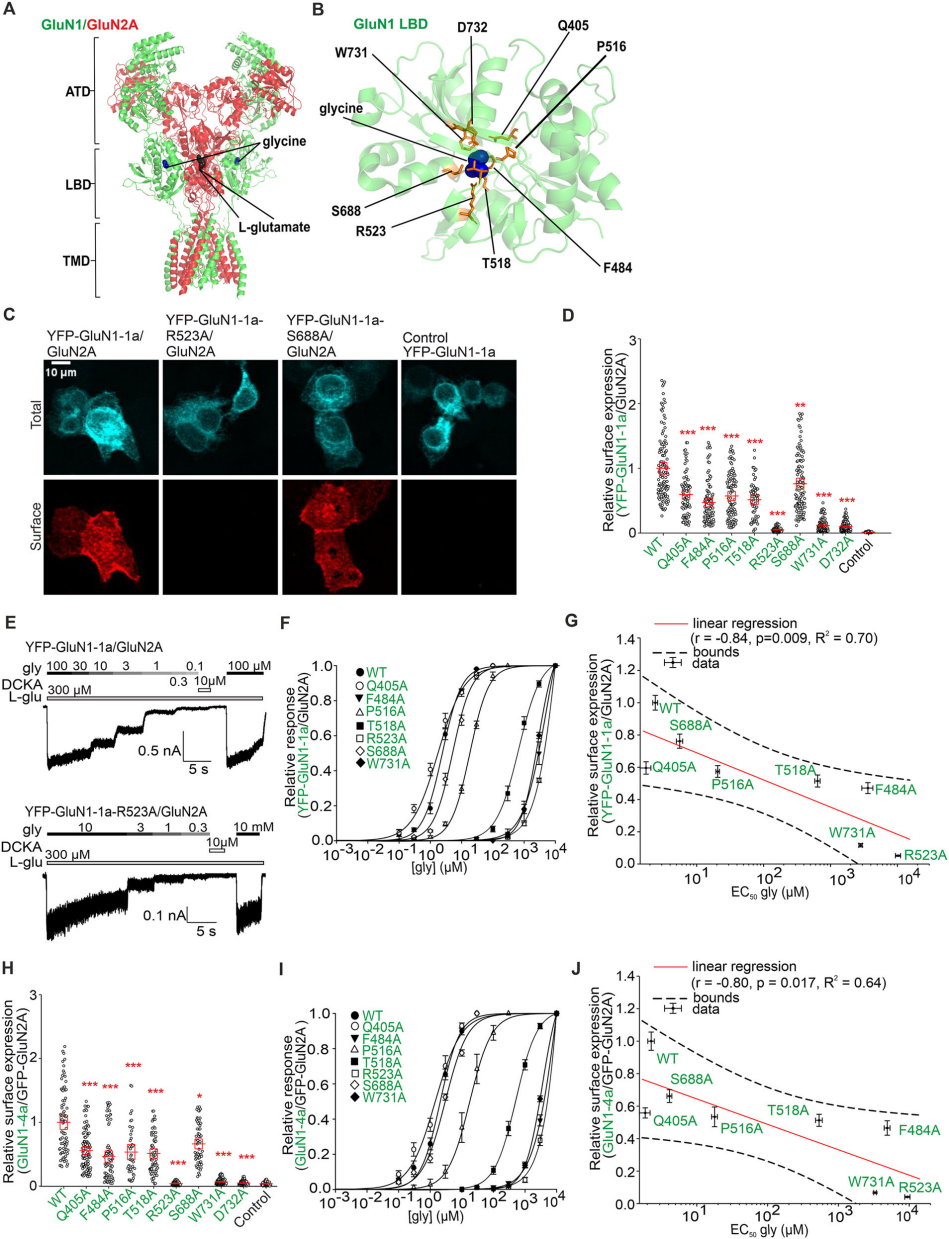
Mutations of residues in the LBD of the GluN1 subunit that interact with glycine affect surface expression and glycine potency of GluN1/GluN2A receptors. ***A***, The structural model of the NMDAR comprises GluN1 (depicted in green) and GluN2A (depicted in red) subunits (based on PDB ID: 7EU7). ***B***, The structural model of the LBD of GluN1 subunit with labeled glycine-interacting amino acids (based on PDB ID, 1PB7). ***C***, Representative images of HEK293 cells transfected with either the YFP-GluN1-1a subunit (WT or mutant variant) alone (negative control) or with the GluN2A subunit. The total and surface signals (top and bottom row, respectively) of YFP-GluN1-1a subunits were labeled using an anti-GFP antibody 24 h after the transfection. ***D***, ***H***, Summary of the relative surface expression of NMDARs consisting of either WT or mutated YFP-GluN1-1a subunit coexpressed with the GluN2A subunit (***D***) and WT or mutated GluN1-4a subunit coexpressed with the GFP-GluN2A subunit (***H***), measured using fluorescence microscopy; *p < 0.05; **p < 0.010; and ***p < 0.001 versus WT; one-way ANOVA. Data points correspond to individual cells (*n* ≥ 56), and the red box plot indicates mean ± SEM. ***E***, Representative whole-cell patch–clamp recordings of HEK293 cells expressing the indicated GluN subunits. Glycine (gly) at the indicated concentrations was applied in the continuous presence of 300 µM l-glutamate (l-glu). ***F***, ***I***, Normalized concentration–response curves for glycine measured from HEK293 cells expressing NMDARs containing YFP-GluN1-1a (***F***) or GluN1-4a subunit variants (***I***) coexpressed with the GluN2A subunit were obtained by fitting the data using Equation 1 (see Materials and Methods); for the summary of fitting parameters, see Table 1. ***G***, ***J***, Correlation of surface expression and glycine EC_50_ values at NMDARs composed of WT or mutated YFP-GluN1-1a subunit (***G***) and WT or mutated GluN1-4a (***J***) subunit expressed together with GFP-GluN2A subunit; data were fitted by linear regression.

*N*-Methyl-d-aspartate receptors (NMDARs) are a family of ionotropic glutamate receptors that play a crucial role in excitatory transmission in the mammalian central nervous system (CNS). Conventional NMDARs are heterotetramers of two GluN1 and two GluN2 subunits in a 1-2-1-2 arrangement. Eight known splice variants of the GluN1 subunit arise from a single gene, and there are four different genes for the GluN2 subunits: GluN2A, GluN2B, GluN2C, and GluN2D (Sanz-Clemente et al., 2012; Vieira et al., 2020; Hansen et al., 2021). Each GluN subunit consists of four membrane helices (M1–M4), an intracellular C-terminal domain (CTD), an extracellular amino-terminal domain (ATD), and an extracellular loop between M3 and M4 (Fig. 1*A*). The ligand-binding domain (LBD) is formed by two segments, S1 and S2 (Traynelis et al., 2010; Paoletti et al., 2013). Critical amino acid residues in the LBDs that are involved either by direct interactions or via water molecules in the interaction with agonists (glycine in the GluN1 subunit and l-glutamate in the GluN2 subunits, respectively) are highly conserved among mammals, highlighting their critical importance for the proper function of NMDARs (Stroebel and Paoletti, 2021).

One of the critical parameters contributing to the physiological or pathophysiological roles of NMDARs is their number on the cell surface, determined by the balance between their exocytosis and endocytosis. Exocytosis (hereafter referred to as “early trafficking”) of NMDARs is regulated at several levels. This includes control over their translation, the proper assembly of functional heterotetramers and maturation in the endoplasmic reticulum (ER), and intracellular trafficking via Golgi apparatus (GA; Meddows et al., 2001; Schüler et al., 2008; Horak et al., 2014; Hanus and Ehlers, 2016). Alternative pathways bypassing the GA have also been identified (Jeyifous et al., 2009). The prevailing hypothesis dealing with the processing of NMDARs in the ER postulates that the ER quality control machinery verifies the sensitivity of NMDARs to their (co)agonists via a common mechanism shared with the α-amino-3-hydroxy-5-methyl-4-isoxazolepropionic acid receptors and kainate receptors (Penn et al., 2008; Coleman et al., 2009, 2010; Scholefield et al., 2019; Horak et al., 2021). However, in the case of GluN1/GluN2 receptors, this hypothesis is supported by a relatively limited number of studies, namely, (1) on the mutant GluN1-D732A/GluN2A receptor, which shows virtually zero surface expression and an extremely high EC_50_ value for glycine due to disruption of direct glycine interaction with GluN1-D732 residue (Kenny et al., 2009), (2) GluN1/GluN2B receptors carrying mutations that disrupt direct (GluN2B-E413A, GluN2B-S690G, GluN2B-R519K) and water (GluN2B-V686A) interaction with l-glutamate or that indirectly lead to changes in EC_50_ values for l-glutamate (GluN2B-F416S; She et al., 2012), and (3) pathogenic variants in the LBDs of the GluN2A and GluN2B subunits, specifically, that disrupt direct (GluN2B-E413G, GluN2A-R518H) and water (GluN2A-G483R, GluN2A-V685G, GluN2A-D731N) interaction with l-glutamate or are present elsewhere in the LBDs (GluN2A-T531M, GluN2B-C461F, GluN2A-I694T, GluN2A-M705V, GluN2A-A716T, GluN2A-A727T, GluN2A-V734L, GluN2A-K772E; Swanger et al., 2016). However, our recent study showed that even a profound reduction in the EC_50_ value for glycine does not lead to a decrease in the surface number of GluN1-S688Y/GluN2 receptors (Skrenkova et al., 2020), supporting further research addressing the roles of LBDs in the early trafficking of GluN1/GluN2 receptors.

We investigated the early trafficking of GluN1/GluN2A receptors by mutagenesis of amino acid residues in the LBDs of GluN1 and GluN2A subunits involved in direct interaction with agonists. Our experiments showed a statistically significant (for alanine substitutions in the GluN1 subunit) and no (for alanine substitution in the GluN2A subunit) correlation between surface expression levels and EC_50_ values for glycine or l-glutamate, respectively. In addition, coexpression of both GluN1 and GluN2A subunits with alanine substitutions additively or synergically altered the surface numbers of GluN1/GluN2A receptors. The experimental and in silico findings from the study of the mutant cycles of the GluN1-S688 and GluN2A-S511 residues revealed that the LBDs of the GluN1 and GluN2A subunits distinctly regulate the early trafficking of GluN1/GluN2A receptors.

## Materials and Methods

### Molecular biology

We used the following DNA vectors carrying genes encoding rat versions of the YFP-GluN1-1a (NP_058706.1), GluN1-4a (NP_001257539.1), GluN2A or GFP-GluN2A (NP_036705), GluN2B or GFP-GluN2B (NP_036706), GluN3A (NP_612555.1), and human GluN2A (hGluN2A, NP_001127879.1) subunits. Human versions of YFP-GluN1-1a (YFP-hGluN1-1a) and GluN1-4a (hGluN1-4a) were prepared as we published previously (Skrenkova et al., 2020). The closed-cleft conformation of LBDs of the GluN1-4a (GluN1-4a-N499C-Q686C) and GFP-GluN2A (GFP-GluN2A-K487C-N687C) subunits was constructed according to previous studies (Blanke and VanDongen, 2008; Kussius and Popescu, 2010; Dai and Zhou, 2016). An ARIAD-mNEONGreen-GluN1-1a construct (ARIAD-mNEON-GluN1-1a) containing the ARIAD sequence (Hangen et al., 2018) followed by a rat version of the GluN1-1a subunit with an inserted mNEONGreen sequence after the 21st amino acid residue was prepared by gene synthesis (Thermo Fischer Scientific) and subsequently subcloned into the pcDNA3 expression vector. A human version of the ARIAD-mNEONGreen-GluN1-1a construct (ARIAD-mNEON-hGluN1-1a) was made by introducing the following amino acid substitutions: N159S, R212K, I267L, and M415L. The specific amino acid substitutions were performed using the QuikChange site-directed mutagenesis kit (Agilent Technologies) and verified by DNA sequencing.

### HEK293 cells and primary hippocampal neurons

Human embryonic kidney 293 (HEK293) cells were grown in Opti-MEM I medium supplemented with 5% fetal bovine serum (FBS; both from Thermo Fischer Scientific) and used in experiments up to Passage 10. For electrophysiology, HEK293 cells in 24-well plates were transfected with a total of 0.9 μg of DNA vectors carrying genes encoding GluN1 and GluN2 subunits and green fluorescent protein (GFP; pQBI 25 vector; for identification of transfected cells; Takara Bio) in a 1:1:1 ratio using 0.9 μl of PolyMag reagent (OZ Biosciences) in 50 μl of Opti-MEM I medium. After 20 min incubation on a magnetic plate, HEK293 cells were trypsinized and resuspended in Opti-MEM I medium containing 1% FBS, 2 mM MgCl_2_, and 10 µM 5,7-dichlorokynurenic acid (DCKA, HelloBio; in the case of NMDARs with mutant GluN2 subunit) or 10 µM d-2-amino-5-phosphonopentanoic acid (D-APV, Hello Bio; in the case of NMDARs with mutant GluN1 subunit) to reduce cell death caused by excitotoxicity. For microscopy, HEK293 cells were grown in 12-well plates and transfected with a total of 0.45 μg of DNA vectors carrying genes encoding GluN1 and GluN2 subunits at a ratio of 1:2 (YFP-/GFP-GluN subunit vs untagged GluN subunit), using 1 μl of Lipofectamine 2000 reagent (Thermo Fischer Scientific) in 50 μl of Opti-MEM I medium. DNA vectors carrying wild-type (WT) or mutant ARIAD-mNEON-GluN1-1a constructs were cotransfected at a 1:2 ratio with untagged GluN2A or GluN3A subunits.

All animal procedures were performed in accordance with the ARRIVE guidelines and the European Commission Council Directive 2010/63/EU for animal experiments. Primary cultures of hippocampal neurons were prepared from Wistar rats of both sexes (at Embryonic Day 18) by dissecting hippocampal neurons in a cold dissection solution consisting of Hanks’ balanced salt solution supplemented with 10 mM HEPES, pH 7.4. Hippocampi were then incubated in a dissection medium containing 0.05% trypsin and 0.1 mg/ml DNase I (Merck) for 20 min at 37°C. Dissociated cells were plated on poly-l-lysine–coated glass coverslips at a density of 2 × 10,000 cells per cm^2^ in a plating medium consisting of minimal essential medium supplemented with 10% heat-inactivated horse serum, N2 supplement (1×), 1 mM sodium pyruvate, 20 mM d-glucose, 25 mM HEPES, and 1% penicillin–streptomycin. After 3 h, the plating medium was replaced entirely with Neurobasal medium enriched with 2% B-27 and 2 mM l-glutamine (all purchased from Thermo Fischer Scientific). Every 3–4 d, ∼50% of the volume of this culture medium was replaced with fresh medium. Hippocampal neurons were transfected at Day 12 in vitro (DIV12) with DNA vectors carrying genes encoding the indicated GluN subunits using Lipofectamine 2000 (Lichnerova et al., 2015).

### Immunofluorescence microscopy

Immunofluorescence labeling was performed 24 h after the completion of transfection of HEK293 cells. Hippocampal neurons were labeled 48 h after the completion of transfection (DIV14). All primary and secondary antibodies were diluted in a blocking solution consisting of PBS and 0.2% (v/v) bovine serum albumin (BSA; Merck). Cells were first washed with ice-cold PBS and incubated for 5 min in a blocking solution on ice. Surface antigens were labeled with primary antibody (rabbit anti-GFP, 1:1,000, Merck) for 15 min on ice, and then cells were washed with ice-cold blocking solution and incubated with secondary antibody (goat anti-rabbit with Alexa Fluor 555, 1:1,000, Thermo Fisher Scientific). Cells were then washed with ice-cold PBS, fixed in 4% paraformaldehyde (PFA) in PBS for 15 min, permeabilized with 0.25% Triton X-100 in PBS for 5 min, and incubated with primary antibody (mouse anti-GFP, 1:1,000, clone N86/38; NeuroMab) and then with secondary antibody (goat anti-mouse conjugated with Alexa Fluor 488, 1:1,000, Thermo Fisher Scientific) to label intracellular antigens.

To examine the internalization rate, surface-expressed NMDARs were labeled as described above. After incubation with primary antibody, HEK293 cells were returned to a conditioned medium without antibody and maintained in a cell culture incubator for 30 min. Subsequently, the cells were washed with PBS, fixed in 4% PFA in PBS for 7 min, and blocked with 0.2% BSA for 5 min. Cells were then incubated with a secondary antibody (goat anti-rabbit conjugated with Alexa Fluor 647, 1:250, Thermo Fisher Scientific). After permeabilization with 0.25% Triton X-100 in PBS, cells were incubated in a blocking solution supplemented with 0.1% Triton X-100 for 30 min, followed by incubation with another secondary antibody (goat anti-rabbit conjugated with Alexa Fluor 555, 1:500, Thermo Fisher Scientific) for 30 min to label internalized NMDARs.

In experiments with ARIAD-mNEON-GluN1-1a constructs, HEK293 cells were first incubated with ARIAD ligand (AL) at a concentration of 1 µM (AL; D/D Solubilizer; Takara Bio) for the indicated times, fixed with PFA for 7 min, labeled using primary antibody (mouse anti-mNEONGreen, 1:1,000, ChromoTek) and secondary antibody (goat anti-mouse conjugated with Alexa Fluor 555, 1:1,000, Thermo Fisher Scientific), and refixed by PFA; intracellular mNEONGreen epitopes in HEK293 cells were not labeled with antibodies. To detect the *cis*-GA structures, we used fixed HEK293 cells labeled with primary antibody (rabbit anti-GM130, 1:1,000, Merck) and secondary antibody (goat anti-rabbit conjugated with Alexa Fluor 555, 1:1,000); labeled cells were then refixed with PFA in PBS for 5 min. Microscopic samples were mounted on glass slides using ProLong antifade reagent (Thermo Fisher Scientific).

Images were captured using either an Olympus FV10i microscope with a 60×/1.35 oil immersion objective (relative surface expression, colocalization with GA) or an Olympus SpinSR10 microscope with a 60×/1.42 oil immersion objective (internalization assay) and analyzed using the ImageJ 1.52N software (National Institutes of Health). In the case of HEK293 cells, total and surface (also the internalized) fluorescence intensity signals were analyzed on whole cells; in the case of hippocampal neurons, 10 separate 5 µm segments of secondary or tertiary dendrites from a single neuron were analyzed. The degree of colocalization of GM130 and mNEONGreen signals was determined as the ratio between the intensity of the mNEONGreen signal colocalizing with the GM130 signal and the intensity of the mNEONGreen signal outside the colocalization region as used previously (Skrenkova et al., 2019). The following competitive antagonists were employed for microscopical experiments: *trans*-2-carboxy-5,7-dichloro-4-phenylaminocarbonylamino-1,2,3,4-tetrahydroquinoline (L-689-560; Tocris Bioscience); 7-chloro-4-hydroxy-3-(3-phenoxy)phenyl-2(1H)-quinolinone (L-701-324; Tocris Bioscience); (*R*)-3-(2-carboxypiperazin-4-yl)propyl-1-phosphonic acid [(R)-CPP; Merck]; [[[(1*S*)-1-(4-bromophenyl)ethyl]amino](1,2,3,4-tetrahydro-2,3-dioxo-5-quinoxalinyl)methyl] phosphonic acid tetrasodium salt (PEAQX; Hello Bio).

### Electrophysiology

Whole-cell current responses of HEK293 cells were recorded by the patch-clamp method ∼24–48 h after completion of transfection using an Axopatch 200B amplifier (Axon Instruments) with series resistance (<10 MΩ) and capacitance compensation (∼80%). Current responses of NMDARs were recorded in a voltage-clamp mode with constant membrane potential held at −60 mV, filtered using an eight-pole Bessel filter (frequency, <2 kHz), sampled at 5 kHz using Digidata 1550 (Molecular Devices), and recorded using pCLAMP 10.7 software (Axon Instruments). Borosilicate glass micropipettes with a tip resistance of ∼4–5 MΩ were prepared using a P-1000 puller (Sutter Instrument) and then filled with an intracellular recording solution containing the following (in mM): 120 gluconic acid, 15 CsCl, 10 BAPTA, 10 HEPES, 3 MgCl_2_, 1 CaCl_2_, and 2 ATP-Mg salts, pH 7.2 (adjusted with CsOH). The standard extracellular recording solution (ECS) contained the following (in mM): 160 NaCl, 2.5 KCl, 10 HEPES, 10 d-glucose, 0.2 EDTA, and 0.7 CaCl_2_, pH 7.3 (adjusted with NaOH). ECS containing the indicated concentrations of l-glutamate and glycine were applied using a multibarrel rapid application system with a time constant of solution exchange around the recorded cell of ∼20 ms (Skrenkova et al., 2020). All electrophysiological recordings were acquired at room temperature. To demonstrate the functionality of GluN1-4a-N499C-Q686C-R523A/GFP-GluN2A, GluN1-4a/GFP-GluN2A-K487C-N687C-Y730A, and GluN1-4a/GFP-GluN2A-K487C-N687C-Y761A receptors, we used a 1 min treatment with 50 mM 2-mercaptoethanol (BME; Merck; Blanke and VanDongen, 2008; Kussius and Popescu, 2010).

The concentration-dependent effects of agonists were analyzed using the following:

$$I=I_{\max}/(1+{\left(EC_{50}/[\mathrm{agonist}]\right)}^{h}),\;(1)$$

where *I*_max_ is the maximal steady-state current amplitude in response to agonist, EC_50_ is the concentration of the agonist eliciting half of the maximal response, [agonist] is the concentration of agonist, and *h* is the apparent Hill coefficient. The approach for estimation of *K*_d_ values is described in Text S1.

### Molecular dynamics (MD) simulations of the mutant cycles of the GluN1-S688 and GluN2A-S511 residues

Complex crystal structures of LBDs of human GluN1/GluN2A receptors were obtained from the RSCB Protein Data Bank (PDB ID, 5KCJ; Hackos et al., 2016). Prior to molecular modeling, the positive allosteric modulator GNE-6901 was removed from the protein-ligand complex. A total of 18 LBD structures were prepared and simulated, including the WT GluN1 subunit along with eight single-point mutants: GluN1-S688A, GluN1-S688P, GluN1-S688C, GluN1-S688N, GluN1-S688E, GluN1-S688K, GluN1-S688W, and GluN1-S688Y. Similarly, the GluN2A subunit was studied in its WT form alongside eight corresponding single-point mutations: GluN2A-S511A, GluN2A-S511P, GluN2A-S511C, GluN2A-S511N, GluN2A-S511E, GluN2A-S511K, GluN2A-S511W, and GluN2A-S511L. Homology models for all selected LBD structures were modeled on a SWISS-MODEL server (Waterhouse et al., 2018) based on UNIPROT targeted sequences (UNIPROT ID, Q05586 for GluN1 and Q12879 for GluN2A). Protonation states of all ionizable residues at physiological pH were assigned for individual LBD variants using the H++ method (Anandakrishnan et al., 2012). Standard AMBER parametrization procedure was used to parametrize glycine and l-glutamate molecules with protonation corresponding to physiological pH. Partial charges were calculated using the Gaussian 16 program (Frisch et al., 2016) using the HF/6-31G* theory level in the gas phase. Parametrized ligands were aligned to precise crystal positions based on the 5KCJ structure.

All MD simulations were carried out using AMBER24 simulation software (Case et al., 2024). Amber ff19SB force field parameters were used for protein description (Tian et al., 2020). All LBD variants were positioned within a cubic simulation box (13 × 13 × 13 nm) and subsequently solvated using the OPC water model (Izadi et al., 2014). Li-Merz ions (Sengupta et al., 2021) were added to replicate the physiological Na^+^/Cl^−^ ion concentration of 150 mM and to ensure system electroneutrality. Prior to the main production run, each system was subjected to energy minimization and gradual thermalization from 10 to 303.15 K. The final production runs of individual LBD variants were conducted in the NVT ensemble with a time step of 2 fs and a total length of simulation time set to 1 µs. The weak-coupling algorithm (Berendsen et al., 1984) maintained the temperature at 310.15 K with a temperature coupling constant of 0.2 ps. During MD simulation, all hydrogen-containing bonds were constrained using the SHAKE algorithm (Ryckaert et al., 1977). A 1.0 nm cutoff was applied to the Lennard-Jones and short-range electrostatic interactions, while long-range electrostatics were handled using particle mesh Ewald summation (Darden et al., 1993). Periodic boundary conditions were applied in all directions of a cubic simulation box. The key structural features of individual simulated systems were analyzed using the *cpptraj* tool (Roe and Cheatham, 2013). The root mean square deviation (RMSD) was used to monitor the overall stability of the receptor by measuring deviations of atomic positions with respect to the initial (crystal) structure. The root mean square fluctuation (RMSF) was used to quantify the flexibility of individual residues with protein sequence to identify regions with significant conformational changes. Furthermore, solvent-accessible surface area (SASA) analysis assessed the extent of solvent exposure and provided insights into the overall compactness of individual LBD variants. To evaluate the energy behind the binding interaction of ligands (glycine or l-glutamate) within the LBDs of the GluN1 and GluN2A subunits, we performed molecular mechanics Poisson–Boltzmann surface area (MM-PBSA) calculations (Miller et al., 2012). All analyses were performed from the last 500 ns of the production simulations.

### Statistical analyses

For microscopical data, statistical analysis was performed for normalized and log-transformed data to stabilize variability against the mean. Data were cleaned of outliers outside the 1.5 times interquartile range and tested for normality using the D'Agostino–Pearson’s test. Statistical significance was tested using a one-factor or two-factor (experiments with ARIAD-mNEON-GluN1-1a construct) ANOVA, followed by Tukey's variant of multiple comparisons via MATLAB (MATLAB, 2022b; using the functions anova1, anovan, and multcompare). Negative controls in the microscopy experiments document the quality of the microscopy staining performed but were not included in the statistical analyses because only one GluN1 or GluN2 subunit was expressed. Differences with a *p* < 0.050 were considered statistically significant; data are presented as mean ± standard error of the mean (SEM). The *rubustfit* function in MATLAB was used to analyze the linear regression; points whose linear regression residuals were ≥2 times the median absolute deviation were identified as outliers. The regression quality was expressed as *R*^2^ (coefficient of determination), and the 95% confidence intervals of the regression line were calculated using the *predint* function (parameter functional, on). Pearson's coefficients of linear correlation (expressed as *r*) and corresponding *p* values were determined to validate the statistical significance of the observed correlations using the *corr* function in MATLAB. If the *p* value was >0.05, we interpreted the correlation as nonsignificant. The strength of the correlation was determined by the absolute value of *r*, classified as follows: very strong (0.80–1.00), strong (0.60–0.79), moderate (0.40–0.59), weak (0.20–0.39), and very weak (0.01–0.19).

## Results

### Alanine substitutions of amino acid residues in the LBD of the GluN1 subunit differentially alter the surface delivery of GluN1/GluN2A receptors

We aimed to determine whether the alanine substitution of amino acid residues in the LBD of the GluN1 subunit that directly interacts with its agonist glycine alters the early trafficking of GluN1/GluN2A receptors. Previous structural studies have shown that within the LBD of the GluN1 subunit, the carboxyl group of glycine interacts with the guanidine group of residue R523, the amino groups of residues T518 and S688, and the hydroxy group of residue S688. The amino group of glycine interacts with residues P516, T518, and D732, the side chain of residue F484 is responsible for the hydrophobic interaction with glycine, and the closed LBD structure is formed by an interdomain interaction between residues Q405, W731, and D732 (Fig. 1*B*; Furukawa and Gouaux, 2003; Inanobe et al., 2005). We first decided to coexpress the WT YFP-GluN1-1a or mutant YFP-GluN1-1a subunits together with the WT GluN2A subunit in HEK293 cells because (1) the GluN1-1a splice variant is retained in the ER in the absence of GluN2 subunits (Huh and Wenthold, 1999), (2) the GluN2A subunit is abundantly represented GluN2 subtype in the adult forebrain (Monyer et al., 1994; Sheng et al., 1994; Maier et al., 2007), (3) the role of LBDs of both the GluN1 or GluN2A subunits in the regulation of early trafficking of the GluN1/GluN2A receptor has not been comprehensively tested, and (4) HEK293 cells do not express endogenous GluN subunits and are commonly used to study the trafficking and functional properties of the NMDARs (Collett and Collingridge, 2004). To minimize the risk of excitotoxic damage caused by the expression of NMDARs, we cultured transfected HEK293 cells in a medium supplemented with 1% FBS, 2 mM Mg^2+^, and 10 µM D-APV or 10 µM DCKA (as described in Materials and Methods). In addition, we used HEK293 cells only up to 10 passages, and each experiment had negative (i.e., YFP-GluN1-1a subunit) and positive (i.e., YFP-GluN1-1a/GluN2A receptor) controls. To accurately detect differences between WT and mutant GluN1/GluN2A receptors, we labeled surface NMDARs by combining a primary polyclonal rabbit anti-GFP antibody with a secondary Alexa Fluor 555-labeled anti-rabbit antibody. Although this combination of primary and secondary antibodies can cluster surface NMDARs, we do not foresee a bias in our microscopic data because this approach (1) provided comparable results to using fluorescently labeled anti-GFP nanobodies in our recent study (Kolcheva et al., 2023) but (2) amplifies the surface signal of NMDARs compared with anti-GFP nanobodies, allowing more accurate detection of differences in surface expression of mutant NMDARs. Initially, we individually replaced the GluN1-Q405, GluN1-F484, GluN1-P516, GluN1-T518, GluN1-R523, GluN1-S688, GluN1-W731, and GluN1-D732 residues, with alanine residues (i.e., a neutral amino acid that should not dramatically alter protein structure) in the YFP-GluN1-1a subunit. Using anti-GFP antibody immunostaining, we measured surface and total expression levels of WT and mutant YFP-GluN1-1a/GluN2A receptors; examples of images of transfected HEK293 cells are shown in Figure 1*C*. We found that the surface expression of YFP-GluN1-1a/GluN2A receptors carrying alanine substitutions showed the following relationship: WT>GluN1-S688A>GluN1-Q405A>GluN1-P516A>GluN1-T518A>GluN1-F484A>GluN1-W731A>GluN1-D732A>GluN1-R523A (Fig. 1*D*). We next performed whole-cell patch–clamp recordings from HEK293 cells expressing WT and mutant YFP-GluN1-1a/GluN2A receptors to learn how alanine substitutions in LBD of the GluN1 subunit alter EC_50_ values for glycine. We elicited the current responses using ECS containing a range of appropriate glycine concentrations (≤10 mM due to potential problems with higher osmolarity) with a fixed concentration of 300 µM l-glutamate and maintaining the membrane potential at −60 mV (Fig. 1*E*). Our electrophysiological experiments showed that HEK293 cells expressing the YFP-GluN1-1a-D732A/GluN2A receptor did not generate current responses at any of the glycine concentrations tested, consistently with our microscopical data showing their disrupted surface expression (Fig. 1*D*). HEK293 cells expressing the remaining mutant YFP-GluN1-1a/GluN2A receptors produced measurable current responses induced by l-glutamate and various glycine concentrations, allowing us to generate concentration–response curves for glycine (Fig. 1*F*). The calculated EC_50_ values for glycine at YFP-GluN1-1a/GluN2A receptors carrying alanine substitutions showed the following relationship: GluN1-Q405A<WT<GluN1-S688A<GluN1-P516A<GluN1-T518A<GluN1-W731A<GluN1-F484A<GluN1-R523A (Table 1). The values of relative surface expression exhibited a very strong and statistically significant negative correlation with the EC_50_ values for glycine (*r* = −0.84; *p* = 0.009), explaining 70% of the variability (*R*^2^ = 0.70; Fig. 1*G*). Thus, our experiments showed that alanine replacements in LBD of the GluN1 subunit mediate alterations in the surface expression of YFP-GluN1-1a/GluN2A receptors which are very strongly correlated with the EC_50_ values for glycine.

**Table 1.** Summary of fitting parameters for steady-state concentration–response curves for glycine measured in HEK293 cells expressing the indicated NMDAR subunits carrying a mutation in the LBD of the GluN1 subunit

Receptor	EC_50_ (μM)	*I*_max_	*h*	*n*	Receptor	EC_50_ (μM)	*I*_max_	*h*	*n*
YFP-GluN1-1a/GluN2A	2.40 ± 0.23	1.00 ± 0.00	1.41 ± 0.03	5	GluN1-4a/GFP-GluN2A	2.27 ± 0.26	1.06 ± 0.03	1.31 ± 0.15	8
YFP-GluN1-1a-Q405A/GluN2A	1.75 ± 0.34	1.01 ± 0.02	1.04 ± 0.08	8	GluN1-4a-Q405A/GFP-GluN2A	1.87 ± 0.36	1.10 ± 0.03	0.95 ± 0.09	5
YFP-GluN1-1a-F484A/GluN2A	3,624.55 ± 787.45	1.23 ± 0.06	1.50 ± 0.13	4	GluN1-4a-F484A/GFP-GluN2A	4,897.02 ± 512.26	1.30 ± 0.08	1.62 ± 0.14	6
YFP-GluN1-1a-P516A/GluN2A	20.61 ± 1.38	1.07 ± 0.02	1.29 ± 0.04	7	GluN1-4a-P516A/GFP-GluN2A	17.87 ± 1.77	1.02 ± 0.01	1.25 ± 0.05	9
YFP-GluN1-1a-T518A/GluN2A	640.72 ± 63.64	1.01 ± 0.00	1.44 ± 0.03	7	GluN1-4a-T518A/GFP-GluN2A	533.11 ± 62.20	1.02 ± 0.01	1.36 ± 0.06	8
YFP-GluN1-1a-R523A/GluN2A	10,261.34 ± 1,052.06	2.02 ± 0.13	1.31 ± 0.08	4	GluN1-4a-R523A/GFP-GluN2A	9,451.12 ± 886.52	1.92 ± 0.14	1.61 ± 0.10	5
YFP-GluN1-1a-S688A/GluN2A	5.48 ± 0.56	1.03 ± 0.01	1.32 ± 0.06	5	GluN1-4a-S688A/GFP-GluN2A	4.12 ± 0.35	1.13 ± 0.04	1.03 ± 0.08	7
YFP-GluN1-1a-W731A/GluN2A	2,824.15 ± 183.58	1.14 ± 0.02	1.61 ± 0.07	5	GluN1-4a-W731A/GFP-GluN2A	3,296.95 ± 165.39	1.14 ± 0.01	1.72 ± 0.02	6
YFP-GluN1-1a-D732A/GluN2A	NR	-	-	12	GluN1-4a-D732A/GFP-GluN2A	NA	-	-	10

The EC_50_, *I*_max_, and *h* values were obtained by fitting the data from individual cells to Equation 1. There was no difference between the YFP-GluN1-1a and GluN1-4a versions; *p* > 0.050, Student's *t* test. NR indicates “not responding,” and NA indicates “not analyzed.”

The CTD splice variants of the GluN1 subunit show remarkable differences in their surface expression. As described above, the GluN1-1a subunit requires the presence of the GluN2 subunit for its release from the ER; in contrast, the GluN1-4a subunit shows robust expression on the cell surface even without the GluN2 subunit (Okabe et al., 1999). Next, we performed an analogous microscopic and electrophysiological analysis in HEK293 cells transfected with WT or untagged GluN1-4a subunits carrying the alanine replacements and the WT GFP-GluN2A subunit as above with the YFP-GluN1-1a/GluN2A receptors. We found the following relationship for the relative surface expression of mutant GluN1-4a/GFP-GluN2A receptors: WT>GluN1-S688A>GluN1-Q405A>GluN1-P516A>GluN1-T518A>GluN1-F484A>GluN1-W731A>GluN1-D732A>GluN1-R523A (Fig. 1*H*). The values of relative surface expression were very strongly and negatively correlated (*r* = −0.80; *p* = 0.017) with the obtained EC_50_ values for glycine (Fig. 1*I*; Table 1), explaining 64% of the variability (*R*^2^ = 0.64; Fig. 1*J*). In the case of HEK293 cells transfected with GluN1-4a-D732A/GFP-GluN2A receptor, we detected only the current responses <100 pA, preventing their detailed concentration-dependent analysis. The measured EC_50_ values for glycine did not differ between the corresponding mutant YFP-GluN1-1a/GluN2A and GluN1-4a/GFP-GluN2A receptors, showing that the presence of GFP or YFP tags as well as differently spliced CTDs of the GluN subunit does not affect the EC_50_ values for glycine (Fig. 1*F*,*I*; Table 1). Our experiments showed that specific alanine substitutions in the LBD of the GluN1 subunit differentially alter the surface trafficking of GluN1/GluN2A receptors, independent of the splice variant of the GluN1 subunit.

### Alanine substitutions of amino acid residues in the LBD of the GluN2A subunit differentially alter the surface expression of GluN1/GluN2A receptors

**Figure 2.**
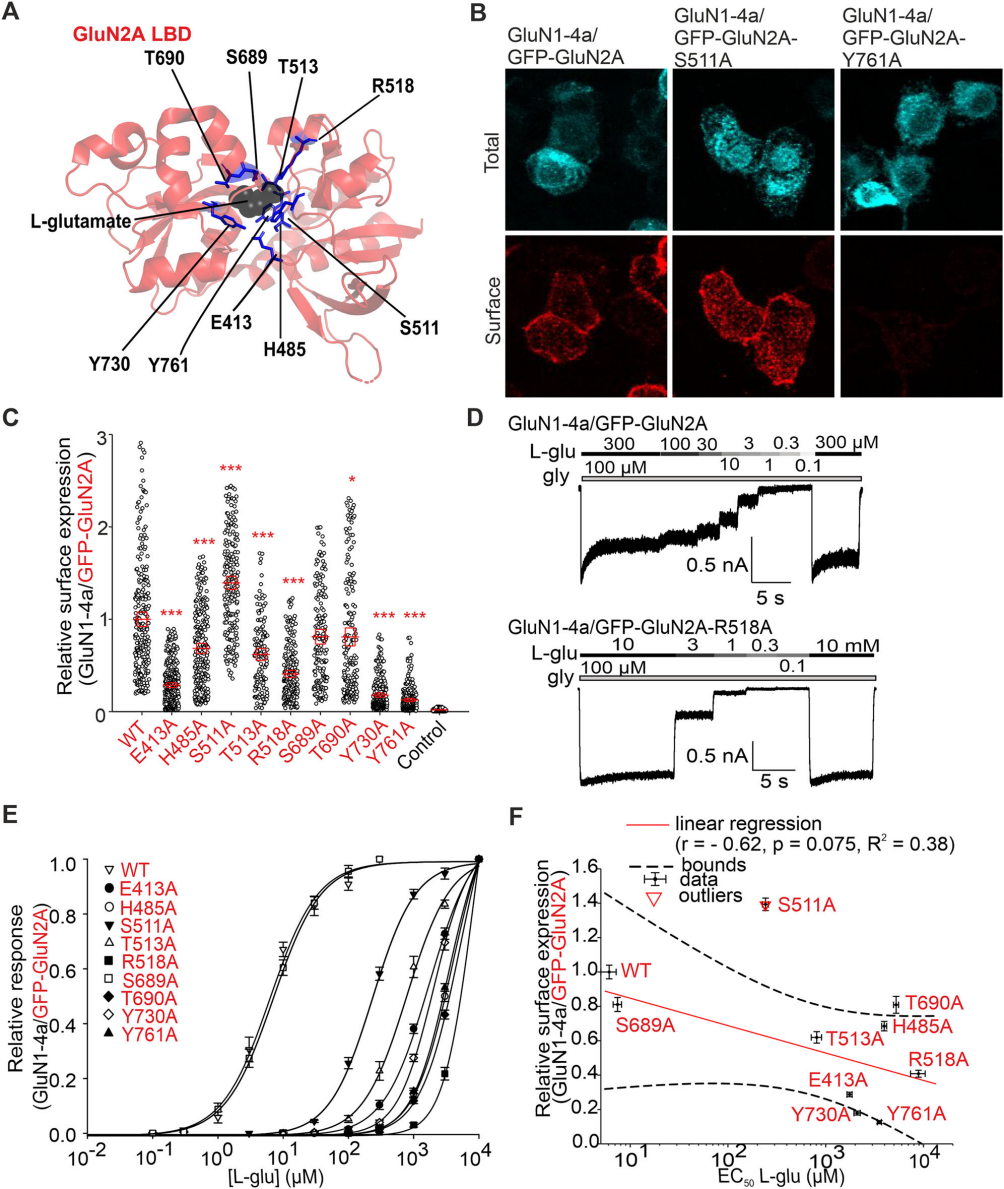
Mutations of residues in LBD of GluN2A subunit interacting with l-glutamate affect surface expression and l-glutamate potency of GluN1/GluN2A receptors. ***A***, The structural model of the LBD of the GluN2A subunit labeled with l-glutamate–interacting amino acids (based on PDB ID, 5I57). ***B***, Representative images of HEK293 cells cotransfected with WT GluN1-4a and WT or mutated GFP-GluN2A subunits. The total and surface signals (top and bottom row, respectively) of GFP-GluN2A subunits were labeled using an anti-GFP antibody 24 h after the transfection. ***C***, Summary of the relative surface expression of NMDARs consisting of either WT or mutated GFP-GluN2A subunit together with WT GluN1-4a subunit, measured using fluorescence microscopy; *p < 0.050 and ***p < 0.001 versus WT; one-way ANOVA. Data points correspond to individual cells (*n* ≥ 136), and the red box plot indicates mean ± SEM. ***D***, Representative whole-cell voltage–clamp recordings of HEK293 cells transfected with the indicated NMDAR subunits. l-Glutamate (l-glu) at the indicated concentrations was applied in the continuous presence of 100 µM glycine (gly). ***E***, Normalized concentration–response curves for l-glutamate measured from HEK293 cells expressing NMDARs containing WT or mutated GluN1-4a/GFP-GluN2A receptors were obtained by fitting the data using Equation 1 (see Materials and Methods); for the summary of fitting parameters, see Table 2. ***F***, Correlation of surface expression and EC_50_ values for l-glutamate at NMDARs composed of WT or mutated GluN1-4a/GFP-GluN2A receptors; data were fitted by linear regression.

We next sought to answer whether alanine substitutions of the amino acid residues involved in its interaction with l-glutamate in LBD of the GluN2A subunit affect the surface expression of GluN1/GluN2A receptors. Previous structural studies with LBD of the GluN2A subunit have shown that the α-carboxyl group of l-glutamate interacts with the side group of residue R518 and with the amino groups of residues S689 and T513 and that the amino group of l-glutamate interacts with the hydroxy groups of residues S511 and T513 and, via the water molecule, with the γ-carboxyl group of residue E413 and the hydroxy group of residue Y761. Also, the γ-carboxyl group of l-glutamate interacts with the amino groups of residues S689 and T690 and the hydroxy group of residue T690. In addition, l-glutamate in LBD is stabilized by hydrophobic interactions with residues H485 and Y730, and the side chains of residues E413 and Y730 form interdomain interactions (Fig. 2*A*; Laube et al., 2004; Furukawa et al., 2005; Chen and Wyllie, 2006; Maier et al., 2007; Jespersen et al., 2014). To reduce the number of the studied mutant NMDARs, the GluN2A-V685, GluN2A-G688, and GluN2A-E691 residues involved in the interaction with l-glutamate via two water molecules were not further tested (Jespersen et al., 2014). Furthermore, the GluN2A-D731 residue was omitted due to conflicting findings in previous studies, which presented varying perspectives on its interaction with l-glutamate, suggesting direct interaction (Jespersen et al., 2014), interaction via water molecules (Hansen et al., 2021), and no interaction with l-glutamate (Furukawa et al., 2005). We cotransfected HEK293 cells with WT or mutant GFP-GluN2A subunits containing the E413A, H485A, S511A, T513A, R518A, S689A, T690A, Y730A, and Y761A mutations, together with the WT GluN1-4a subunit, as this GluN subunit combination allows more straightforward electrophysiological characterization of mutant NMDARs with reduced surface expression (as shown in Fig. 1). We immunofluorescently labeled HEK293 cells expressing WT and mutant GluN1-4a/GFP-GluN2A receptors using an anti-GFP antibody (Fig. 2*B*); our subsequent microscopical analysis showed the following trend in their surface expression: GluN2A-S511A>WT>GluN2A-S689A>GluN2A-T690A>GluN2A-H485A>GluN2A-T513A>GluN2A-R518A>GluN2A-E413A>GluN2A-Y730A>GluN2A-Y761A (Fig. 2*C*). Then, we elicited the current responses by application of l-glutamate in the appropriate concentration range (10 mM) in the continuous presence of 100 µM glycine (Fig. 2*D*). The analysis of the concentration–response curves for l-glutamate at GluN1-4a/GFP-GluN2A receptors showed the following trend for the EC_50_ values: WT<GluN2A-S689A<GluN2A-S511A<GluN2A-T513A<GluN2A-E413A<GluN2A-Y730A<GluN2A-Y761A<GluN2A-H485A<GluN2A-T690A<GluN2A-R518A (Fig. 2*E*; Table 2). There was no correlation (*r* = −0.62; *p* = 0.075; *R*^2^ = 0.38) between the values of the relative surface expression and EC_50_ values for l-glutamate (Fig. 2*F*). The values obtained for the GluN1/GluN2A-S511A receptor were identified as an outlier based on the statistical test described in the Materials and Methods section. These results showed that alanine substitutions in the LBD of the GluN2A subunit regulate the surface expression of GluN1/GluN2A receptors independently of their EC_50_ values for l-glutamate.

**Table 2.** Summary of fitting parameters for steady-state concentration–response curves for l-glutamate effect measured in HEK293 cells expressing the indicated NMDAR subunits carrying a mutation in the LBD of GluN2A subunit

Receptor	EC_50_ (μM)	*I*_max_	*h*	*n*
GluN1-4a/GFP-GluN2A	6.10 ± 0.70	0.96 ± 0.01	1.34 ± 0.04	5
GluN1-4a/GFP-GluN2A-E413A	1,754.81 ± 114.61	1.11 ± 0.01	1.27 ± 0.05	6
GluN1-4a/GFP-GluN2A-H485A	3,969.98 ± 253.57	1.25 ± 0.03	1.50 ± 0.04	6
GluN1-4a/GFP-GluN2A-S511A	235.41 ± 18.32	1.00 ± 0.02	1.35 ± 0.03	6
GluN1-4a/GFP-GluN2A-T513A	809.56 ± 109.74	1.02 ± 0.01	1.33 ± 0.03	5
GluN1-4a/GFP-GluN2A-R518A	8,895.17 ± 1,702.60	1.83 ± 0.29	1.96 ± 0.15	5
GluN1-4a/GFP-GluN2A-S689A	7.42 ± 0.82	1.01 ± 0.02	1.17 ± 0.05	5
GluN1-4a/GFP-GluN2A-T690A	5,254.10 ± 339.72	1.40 ± 0.04	1.45 ± 0.05	7
GluN1-4a/GFP-GluN2A-Y730A	2,099.49 ± 170.86	1.09 ± 0.02	1.58 ± 0.07	6
GluN1-4a/GFP-GluN2A-Y761A	3,542.66 ± 173.32	1.20 ± 0.03	1.58 ± 0.10	8

The EC_50_, *I*_max_, and *h* values were obtained by fitting the data from individual cells to Equation 1.

### Synchronized release from the ER showed that alanine substitutions in LBDs alter the early trafficking of GluN1/GluN2A receptors

**Figure 3.**
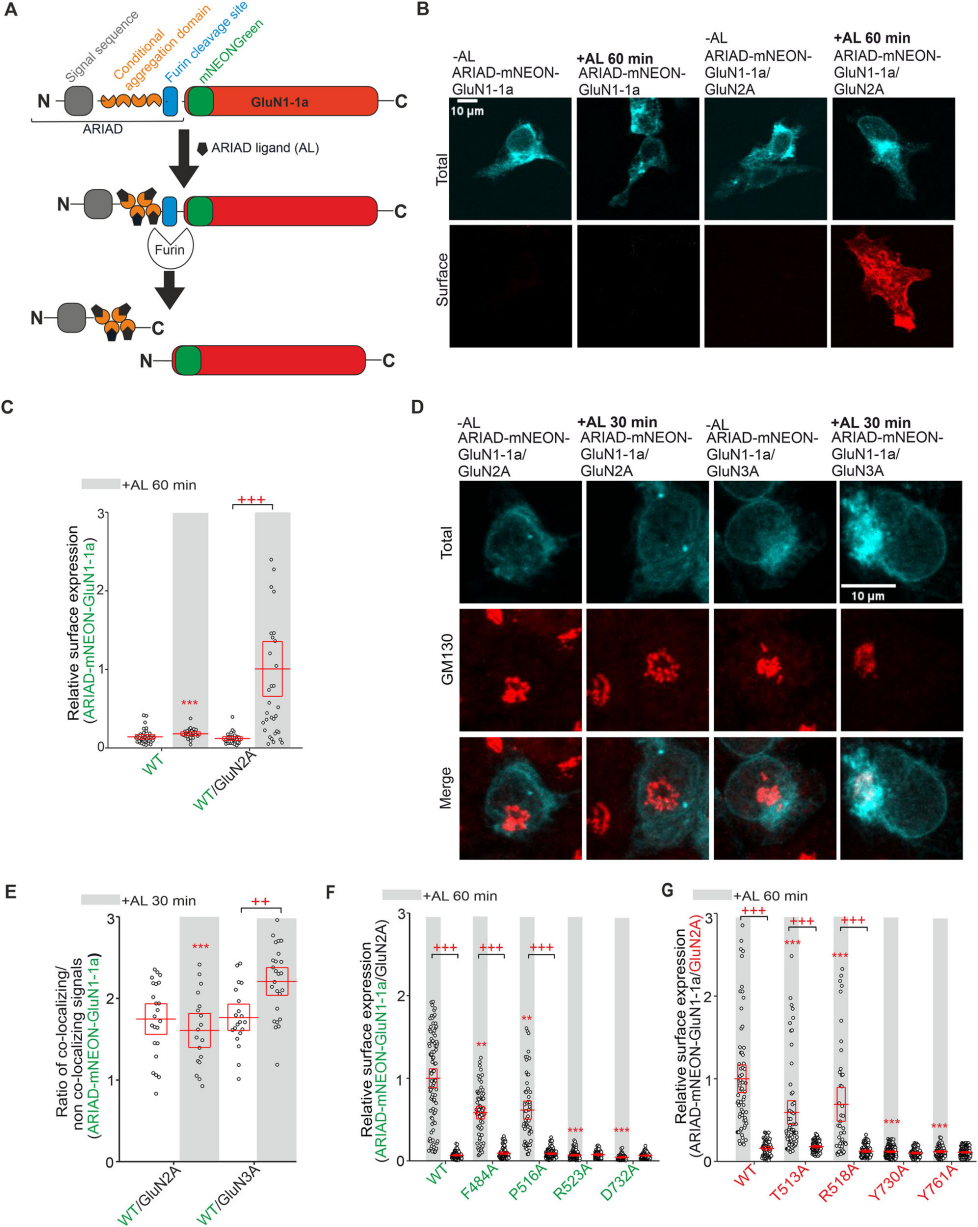
Alanine substitutions in the LBDs of both GluN1 and GluN2A subunits alter the early trafficking of NMDARs. ***A***, Schematic representation of ARIAD-mNEON-GluN1-1a construct with a signal sequence (gray), conditional aggregation domain (orange), furin cleavage site (blue), mNEONGreen [green; mNEON sequence was inserted after the 21st amino acid residue of the GluN1-1a subunit (red)]. Upon the addition of the AL to the cells, AL binds to the conditional aggregation domain, leading to a conformational change and release of the ARIAD-mNEON-GluN1-1a construct from the ER, followed by cleavage of the ARIAD sequence by the protease furin. See also the Materials and Methods section. ***B***, Representative images of HEK293 cells expressing ARIAD-mNEON-GluN1-1a construct either alone or with the GluN2A subunit at 0 min (without AL) and 60 min after adding AL. The total and surface signals (top and bottom row, respectively) of ARIAD-mNEON-GluN1-1a constructs were labeled using an anti-mNEONGreen antibody 24 h after the transfection. The representative images of GluN1-4a/ARIAD-mNEON-GluN2A receptor are shown in Figure S1. ***C***, Summary of relative surface expression of NMDARs containing WT or mutated ARIAD-mNEON-GluN1-1a constructs together with GluN2A subunit in the absence or presence (60 min) of AL, measured using fluorescence microscopy; ***p < 0.001 for differences between ARIAD-mNEON-GluN1-1a/GluN2A in the presence of AL and ARIAD-mNEON-GluN1-1a expressed alone; +++*p* < 0.001 for differences between presence and absence of AL; two-way ANOVA. Data points correspond to individual cells (*n* ≥ 26), and the red box plot indicates mean ± SEM. See Figure S2, *A* and *B*, for a summary of the relative surface expression of the time points for the ARIAD-mNEON-GluN1-1a construct coexpressed with GluN2A or GluN3A subunits. ***D***, Representative microscopy images of the HEK293 cells cotransfected with ARIAD-mNEON-GluN1-1a construct and GluN2A or GluN3A subunits in the absence or presence (30 min) of AL; the anti-GM130 antibody was used to label the GA. ***E***, Summary of the average intensity of ARIAD-mNEON-GluN1-1a subunit signal colocalized with GM130 over the average intensity of ARIAD-mNEON-GluN1-1a subunit signal outside the GM130 signal, calculated for the indicated NMDAR combinations; ***p < 0.001 for differences between ARIAD-mNEON-GluN1-1a/GluN3A receptors in the presence of AL and ARIAD-mNEON-GluN1-1a/GluN2A receptors; ++*p* < 0.01 for differences between the presence and absence of AL; two-way ANOVA. Data points correspond to individual cells (*n* ≥ 18), and the red box plot indicates mean ± SEM. See Figure S3 for a summary of the time points for colocalized ARIAD-mNEON-GluN1-1a construct coexpressed with GluN2A subunits. ***F***, ***G***, Summary of relative surface expression of NMDARs containing WT or mutated ARIAD-mNEON-GluN1-1a subunits coexpressed with WT GluN2A subunit (***F***) or WT ARIAD-mNEON-GluN1-1a subunit coexpressed with WT or mutated GluN2A subunit (***G***), measured in the absence or presence (60 min) of AL, using fluorescence microscopy; **p < 0.010 and ***p < 0.001 for differences between WT and mutated ARIAD-mNEON-GluN1-1a/GluN2A receptors in the presence of AL; +++*p* < 0.001 for differences between the absence and presence of AL; two-way ANOVA. Data points correspond to individual cells (*n* ≥ 55), and the red box plot indicates mean ± SEM. Different internalization rates did not affect the surface expression of GluN1/GluN2A-S511A receptors; see representative images of internalization assay with WT GluN1-4a/GFP-GluN2A and GluN1-4a/GFP-GluN2A-S511A receptors (Fig. S4*A*) and the subsequent quantification of surface and internalized GFP signals (Fig. S4*B*).

We further used the ARIAD system, which allows the synchronized release of membrane proteins from the ER by the addition of the AL (Fig. 3*A*; Hangen et al., 2018) to test the hypothesis that changes in surface expression of mutant GluN1/GluN2A receptors are caused at the level of early trafficking. We created ARIAD-mNEON-GluN1-1a and ARIAD-mNEON-GluN2A constructs and expressed them separately in HEK293 cells. Unfortunately, HEK293 cells expressing the ARIAD-mNEON-GluN2A construct showed a distinct surface signal detected by the anti-mNEONGreen antibody 24 h after transfection, even without adding AL (0 min; Fig. S1). In contrast, HEK293 cells expressing the ARIAD-mNEON-GluN1-1a construct showed no surface signal under control conditions (0 min) or 60 min after adding AL (Fig. 3*B*). Cotransfection of HEK293 cells with the ARIAD-mNEON-GluN1-1a construct and the WT GluN2A subunit resulted in robust surface expression of the mNEON-GluN1-1a/GluN2A receptor 60 min after addition of AL but not under control conditions (0 min), confirming the functionality of the ARIAD-mNEON-GluN1-1a construct for studying early trafficking of GluN1/GluN2A receptors (Fig. 3*C*; time points 0, 30, 60, and 120 min after the addition of AL shown in Fig. S2*A* and for ARIAD-mNEON-GluN1/GluN3A receptor in Fig. S2*B*). Given that only a smaller fraction of functional GluN1/GluN2A receptors likely leave the ER structures in HEK293 cells (Huh and Wenthold, 1999; Hawkins et al., 2004), their colocalization with GA is used as a measure to quantify the early trafficking of NMDAR (She et al., 2012; Skrenkova et al., 2019). Therefore, we cotransfected HEK293 cells with the ARIAD-mNEON-GluN1-1a construct, in combination with the WT GluN2A subunit (or with the WT GluN3A subunit as a positive control) and labeled GA structures on fixed cells with anti-GM130 antibody at 0 and 30 min after addition of AL (Fig. 3*D*). Consistent with our previous experiments with GluN1/GFP-GluN3A receptors (Skrenkova et al., 2019), our microscopical analysis showed that mNEON-GluN1-1a/GluN3A receptor exhibited a higher rate of colocalization with GA structures 30 min after addition of AL compared with negative control (0 min). In contrast, mNEON-GluN1-1a/GluN2A receptors did not show a different rate of colocalization with GA structures 30 min after the addition of AL compared with negative control (0 min; Fig. 3*E*); we obtained the same conclusion in another experiment involving time points 0, 15, 30, 45, and 60 min after the addition of AL (Fig. S3). One possible explanation is that GluN1/GluN2A receptors can bypass GA via an unconventional pathway during early trafficking to the cell surface (see Discussion). Therefore, we did not analyze the colocalization of mutant GluN1/GluN2A receptors with GA structures to measure their early trafficking, and we focused on characterizing their surface expression 60 min after adding AL.

To test whether mutant GluN1/GluN2A receptors exhibit altered early trafficking, we introduced four alanine substitutions into the ARIAD-mNEON-GluN1-1a construct, which caused the most pronounced (∼95% GluN1-R523A and ∼90% GluN1-D732A) and moderate (∼53% GluN1-F484A and ∼43% GluN1-P516A) reductions in surface expression of GluN1/GluN2A receptors (Fig. 1). Consistent with our previous results, GluN1-R523A and GluN1-D732A mutations eliminated surface expression (∼7% for GluN1-R523 and ∼4% for GluN1-D732A). In contrast, both GluN1-F484A and GluN1-P516A mutations caused ∼42% resp. ∼39% reduction in surface expression of mNEON-GluN1-1a/GluN2A receptors measured 60 min after addition of AL (Fig. 3*F*). For further analysis using the ARIAD system, we selected, based on previous experiments, four alanine substitutions in the LBD of the GluN2A subunit, two that caused the most pronounced (∼82% GluN2A-Y730A and ∼87% GluN2A-Y761A) and two that showed moderate (∼38% GluN2A-T513A and ∼59% GluN2A-R518A) reductions in surface expression of GluN1/GluN2A receptors (Fig. 2). Our analysis confirmed that the GluN2A-Y730A and GluN2A-Y761A mutations were essentially eliminated (both ∼12%) and that the GluN2A-T513A and GluN2A-R518A mutations caused a ∼41% resp. ∼31% reduction in surface expression of mNEON-GluN1-1a/GluN2A receptors in HEK293 cells at 60 min after addition of AL (Fig. 3*G*). Interestingly, the GluN1/GluN2A-S511A receptor exhibited increased levels of relative surface expression compared with the WT GluN1/GluN2A receptor (Fig. 2*C*). We next performed an internalization assay with a primary anti-GFP antibody on the HEK239 cells transfected with the WT GluN1-4a/GFP-GluN2A and GluN1-4a/GFP-GluN2A-S511A receptors; representative images are shown in Figure S4*A*. Consistent with our data described above, the GluN1-4a/GFP-GluN2A-S511A receptor exhibited increased surface expression levels, but their relative internalization rate was unaltered compared with WT GluN1-4a/GFP-GluN2A receptor (Fig. S4*B*). This indicates that the increased surface expression of the GluN1/GluN2A-S511A receptor was not mediated by an altered internalization rate. Therefore, we suggest that the observed decrease in surface expression of GluN1/GluN2A receptors with alanine substitutions in the LBDs of both GluN1 and GluN2A subunits is due to their altered early trafficking, likely during ER processing.

### Alanine substitutions in the LBDs of the GluN1 and GluN2A subunits independently affect the surface expression of GluN1/GluN2A receptors, regardless of the presence of the artificial disulfide bonds that create the close conformations of the LBDs

**Figure 4.**
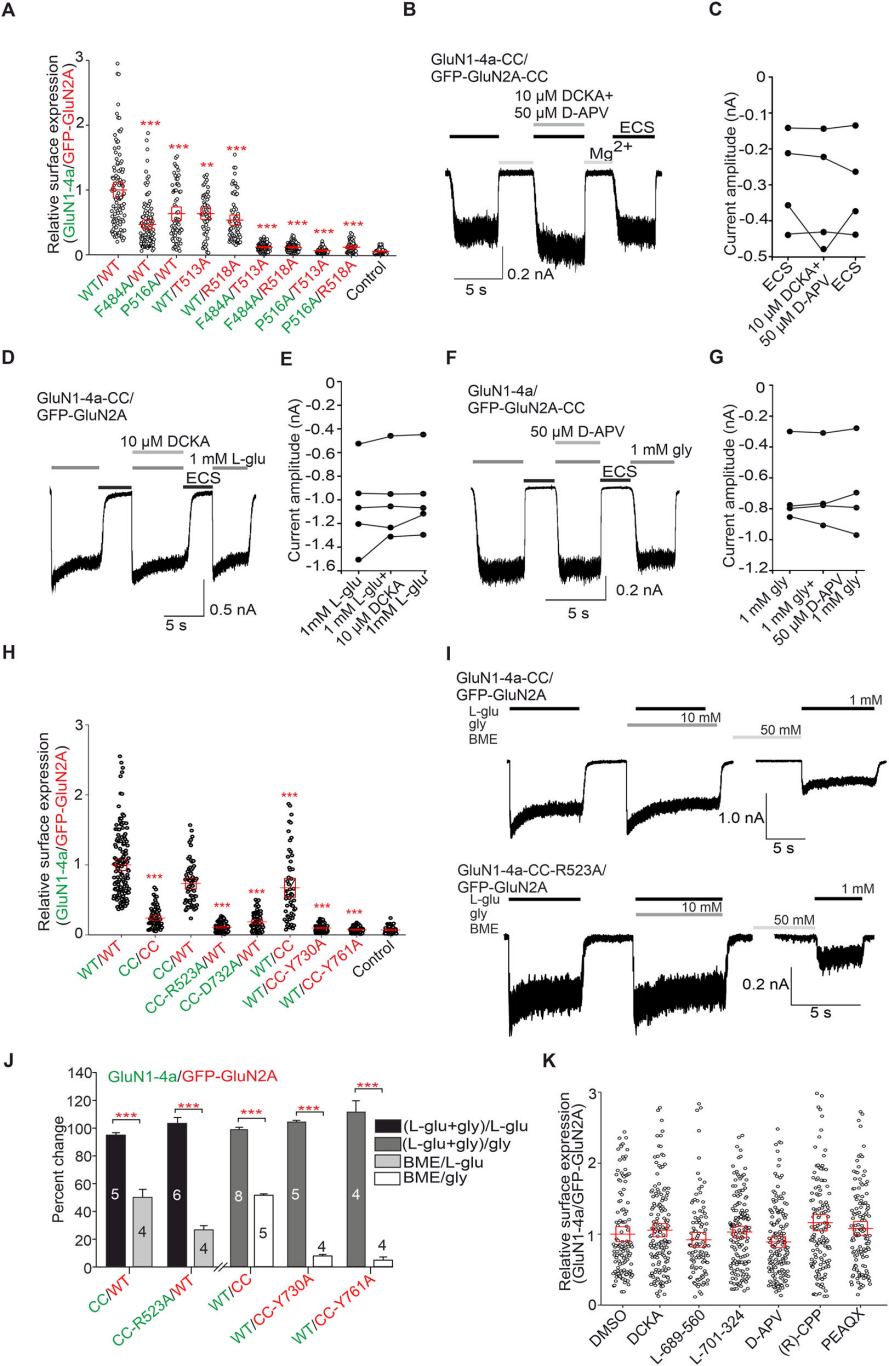
Alanine substitutions in the LBDs of GluN1 and GluN2A subunits additively or synergically regulate the early trafficking of GluN1/GluN2A receptors. A, Summary of the relative surface expression of NMDARs containing mutations within LBDs of both GluN1-4a and GFP-GluN2A subunits, measured using fluorescence microscopy; **p < 0.01 and ***p < 0.001 versus WT; one-way ANOVA. Data points correspond to individual cells (n ≥ 55), and the red box plot indicates mean ± SEM. B, D, F, Representative whole-cell patch–clamp recordings of GluN1-4a-N499C-Q686C/GFP-GluN2A-K487C-N687C (B), GluN1-4a-N499C-Q686C/GFP-GluN2A (D), and GluN1-4a/GFP-GluN2A-K487C-N687C (F) receptors expressed in the HEK293 cells. The current responses were elicited under the specified conditions. ECS denotes Mg2+-free extracellular solution, l-glu represents l-glutamate (1 mM), and gly indicates glycine (1 mM). As indicated, 10 µM 5,7-DCKA and 50 µM D-APV were applied. C, E, G, Comparison of current response amplitudes evoked by ECS without Mg2+ (C), 1 mM l-glutamate plus 10 µM DCKA (E), and 1 mM glycine plus 50 µM D-APV (G). H, Summary of relative surface expression for NMDARs composed of GluN1-4a and GFP-GluN2A subunits with the closed-cleft (CC) conformation of LBDs in combination with selected alanine substitutions, measured using fluorescence microscopy; ***p < 0.001 versus WT; one-way ANOVA. Data points correspond to individual cells (n ≥ 54), and the red box plot indicates mean ± SEM. I, Representative whole-cell patch–clamp recordings from the HEK293 cells expressing the indicated GluN subunits. Glycine (gly) and/or l-glutamate (l-glu) were applied as indicated at a concentration of 1, and 50 mM BME was applied 1 min before the second application of gly or l-glu. J, Summary of the electrophysiological data analysis shown in I; the graph shows the percentage change of the peak current amplitudes induced by indicated combinations of (co-)agonists before and after BME treatment; CC indicates the closed-cleft configuration of GluN1-4a or GluN2A subunits; ***p < 0.001 versus the current response after BME treatment; two-way ANOVA. K, Summary of the effect of indicated competitive antagonists on surface expression of WT GluN1-4a/GFP-GluN2A receptors, measured using fluorescence microscopy; p > 0.05; one-way ANOVA. The abbreviations are as follows: L-689,560, 4-trans-2-carboxy-5,7-dichloro-4-phenylaminocarbonylamino-1,2,3,4-tetrahydroquinoline; L-701,324, 7-chloro-4-hydroxy-3-(3-phenoxy)phenyl-2(1H)-quinolone; PEAQX, 5-phosphonomethyl-1,4-dihydroquinoxaline-2,3-dione; CPP, 4-(3-phosphonopropyl) pizerazine-2-carboxylic acid. Data points correspond to individual cells (n ≥ 107), and the red box plot indicates mean ± SEM.

We then investigated whether alanine substitutions in LBDs of GluN1 and GluN2A subunits are sensed independently by cellular control mechanisms. Therefore, we cotransfected HEK293 cells with a combination of WT and mutant GluN1-4a (containing F484A or P516A mutations) and GFP-GluN2A (containing T513A or R518A mutations) subunits. Our analysis showed that in all cases, the combination of two alanine substitutions (GluN1-4a-F484A/GFP-GluN2A-T513A, GluN1-4a-F484A/GFP-GluN2A-R518A, GluN1-4a-P516A/GFP-GluN2A-T513A, and GluN1-4a-P516A/GFP-GluN2A-R518A) caused reduced surface expression of the GluN1-4a/GFP-GluN2A receptors (∼90%) compared with the GluN1-4a/GFP-GluN2A receptors containing only single alanine substitutions (Fig. 4*A*). Thus, our experiments showed that structural changes in the LBDs of GluN1 and GluN2A subunits additively or synergistically regulate the early trafficking of GluN1/GluN2A receptors and are likely recognized independently by control mechanisms.

Previous studies have found that the introduction of specific cysteine residues into LBDs in the GluN1 (N499C-Q686C) and GluN2A (K487C-N687C) subunits leads to the closed-cleft conformation of the LBDs (Blanke and VanDongen, 2008; Kussius and Popescu, 2010; Dai and Zhou, 2016). We first verified by electrophysiological measurements that GluN1-4a-N499C-Q686C/GFP-GluN2A-K487C-N687C receptor expressed in HEK293 cells generate current responses elicited by ECS application in the absence of glycine and l-glutamate also in the presence of the competitive antagonists (10 µM DCKA and 50 µM D-APV; Fig. 4*B*,*C*). Subsequently, we found that HEK293 cells expressing GluN1-4a-N499C-Q686C/GFP-GluN2A receptor produced current responses in the presence of 1 mM l-glutamate only, which were insensitive to 10 µM DCKA (Fig. 4*D*,*E*). In contrast, HEK293 cells expressing GluN1-4a/GFP-GluN2A-K487C-N687C receptor had current responses elicited by 1 mM glycine only, which were insensitive to 50 µM D-APV (Fig. 4*F*,*G*).

Subsequent microscopical experiments showed that GluN1-4a-N499C-Q686C/GFP-GluN2A-K487C-N687C receptor exhibit profoundly reduced surface expression (∼76%), the GluN1-4a-N499C-Q686C/GFP-GluN2A receptor showed a tendency in reduction (∼27%), and GluN1-4a/GFP-GluN2A-K487C-N687C receptor showed a decrease (∼33%) in surface expression compared with WT GluN1-4a/GFP-GluN2A receptor (Fig. 4*H*). These data support the conclusion that the LBDs of GluN1 and GluN2A subunits additively or synergistically regulate the early trafficking of GluN1/GluN2A receptors. We next asked whether introducing alanine substitutions in the LBDs of the GluN1 (R523A and D732A) or GluN2A (Y730A and Y761A) subunits, which caused a profound reduction in surface expression of GluN1/GluN2A receptors, also affect surface expression of GluN1/GluN2A receptors with closed-cleft conformation of LBD. Our microscopical analysis showed that single alanine substitutions in the closed-cleft conformation of LBDs of both GluN1 and GluN2A subunits, i.e., in the case of GluN1-4a-N499C-Q686C-R523A/GFP-GluN2A, GluN1-4a-N499C-Q686C-D732A/GFP-GluN2A, GluN1-4a/GFP-GluN2A-K487C-N687C-Y730A, and GluN1-4a/GFP-GluN2A-K487C-N687C-Y761A receptors (Fig. 4*H*), induced a profound decrease of their surface expression, comparable to the decline observed for GluN1-4a/GFP-GluN2A receptors carrying only the corresponding alanine substitutions. Based on previous protocols (Blanke and VanDongen, 2008; Kussius and Popescu, 2010), we next aimed to verify the functionality and the formation of the artificial disulfide bonds in the GluN1-4a-N499C-Q686C-R523A/GFP-GluN2A, GluN1-4a/GFP-GluN2A-K487C-N687C-Y730A, and GluN1-4a/GFP-GluN2A-K487C-N687C-Y761A receptors (GluN1-4a-N499C-Q686C-D732A/GFP-GluN2A receptor was not examined as we observed only small current amplitudes of the GluN1-D732A/GluN2A receptor expressed in the HEK293 cells). We induced the current responses using 1 mM l-glutamate (GluN1-4a-N499C-Q686C-R523A/GFP-GluN2A) or 1 mM glycine (GluN1-4a/GFP-GluN2A-K487C-N687C-Y730A and GluN1-4a/GFP-GluN2A-K487C-N687C-Y761A) and using both 1 mM l-glutamate and 1 mM glycine, which was followed by 1-min-long application of 50 mM BME (Fig. 4*I*). These experiments showed that all three tested mutant GluN1/GluN2A receptors produced detectable current responses induced by 1 mM l-glutamate or 1 mM glycine, which were not altered by the coapplication of both 1 mM l-glutamate and 1 mM glycine, ruling out the possibility that the artificial disulfide bonds are formed in only a small subset of surface GluN1/GluN2 receptors. In addition, the current responses were reduced after preincubation with BME, which is in agreement with previous experiments at WT NMDARs (Blanke and VanDongen, 2008; Kussius and Popescu, 2010). Notably, 50 mM BME tended to reduce the current amplitudes more at the mutated (GluN1-4a-N499C-Q686C-R523A/GFP-GluN2A, GluN1-4a/GFP-GluN2A-K487C-N687C-Y730A, GluN1-4a/GFP-GluN2A-K487C-N687C-Y761A) than at the “nonmutated” (GluN1-4a-N499C-Q686C/GFP-GluN2A, GluN1-4a/GFP-GluN2A-K487C-N687C) NMDARs containing artificial disulfide bonds, further supporting the existence of artificial disulfide bonds in GluN1-4a-N499C-Q686C-R523A/GFP-GluN2A, GluN1-4a/GFP-GluN2A-K487C-N687C-Y730A, and GluN1-4a/GFP-GluN2A-K487C-N687C-Y761A receptors expressed on the cell surface of the HEK293 cells (Fig. 4*J*). These experiments demonstrated that the control mechanisms perceive alanine substitutions regardless of the closed-cleft conformation of LBDs of both GluN1 and GluN2A subunits.

Finally, we investigated whether the presence of selected competitive antagonists affects the surface expression of GluN1/GluN2A receptors. Specifically, we used the commonly used DCKA and two highly potent antagonists, L-689-560 and L-701-324, acting via the GluN1 subunit, and the widely used D-APV and two highly potent antagonists, (R)-CPP and PEAQX, with high selectivity for the GluN2A subunit (Traynelis et al., 2010). We cultured the HEK293 cells expressing WT GluN1-4a/GFP-GluN2A receptor for 36 h after the completion of the transfection in the presence of 300 µM of competitive antagonists, as higher concentrations could mediate toxic effects on HEK293 cells (data not shown). We did not observe any differences in the surface expression level of the WT GluN1-4a/GFP-GluN2A receptor in the presence of competitive antagonists (Fig. 4*K*). Using a chemical database (available at URL https://pubchem.ncbi.nlm.nih.gov/), we found the following predicted logP (octanol–water ratio; XLogP3-AA) values: DCKA (2.5); L-689-560 (3.5); L-701-324 (4.5); D-APV (−5.3); (R)-CPP (−6.8); and PEAQX (−1.6). The examined competitive antagonists acting at the GluN1 subunit (DCKA, L-689-560, and L-701-324) thus exhibit the optimal range of membrane permeability (>1; Soliman et al., 2021). Conversely, all the used competitive antagonists acting at the GluN2A subunit [D-APV, (R)-CPP, and PEAQX] are hydrophilic and likely did not reach the ER in our experiments. Thus, our data suggest that quality control mechanisms in the ER are not affected by the presence of competitive antagonists acting at least at the GluN1 subunit. Future experiments are needed to assess the precise concentrations of competitive antagonists in the ER. Nevertheless, our experiments showed that long-term incubation with a panel of competitive antagonists did not alter the surface expression of the GluN1/GluN2A receptor; this supports the use of competitive antagonists in cell-based experiments to reduce NMDAR-induced excitotoxicity.

### The effect of selected alanine substitutions in LBDs on surface expression is conserved for GluN1/GluN2A and GluN1/GluN2B receptors

**Figure 5.**
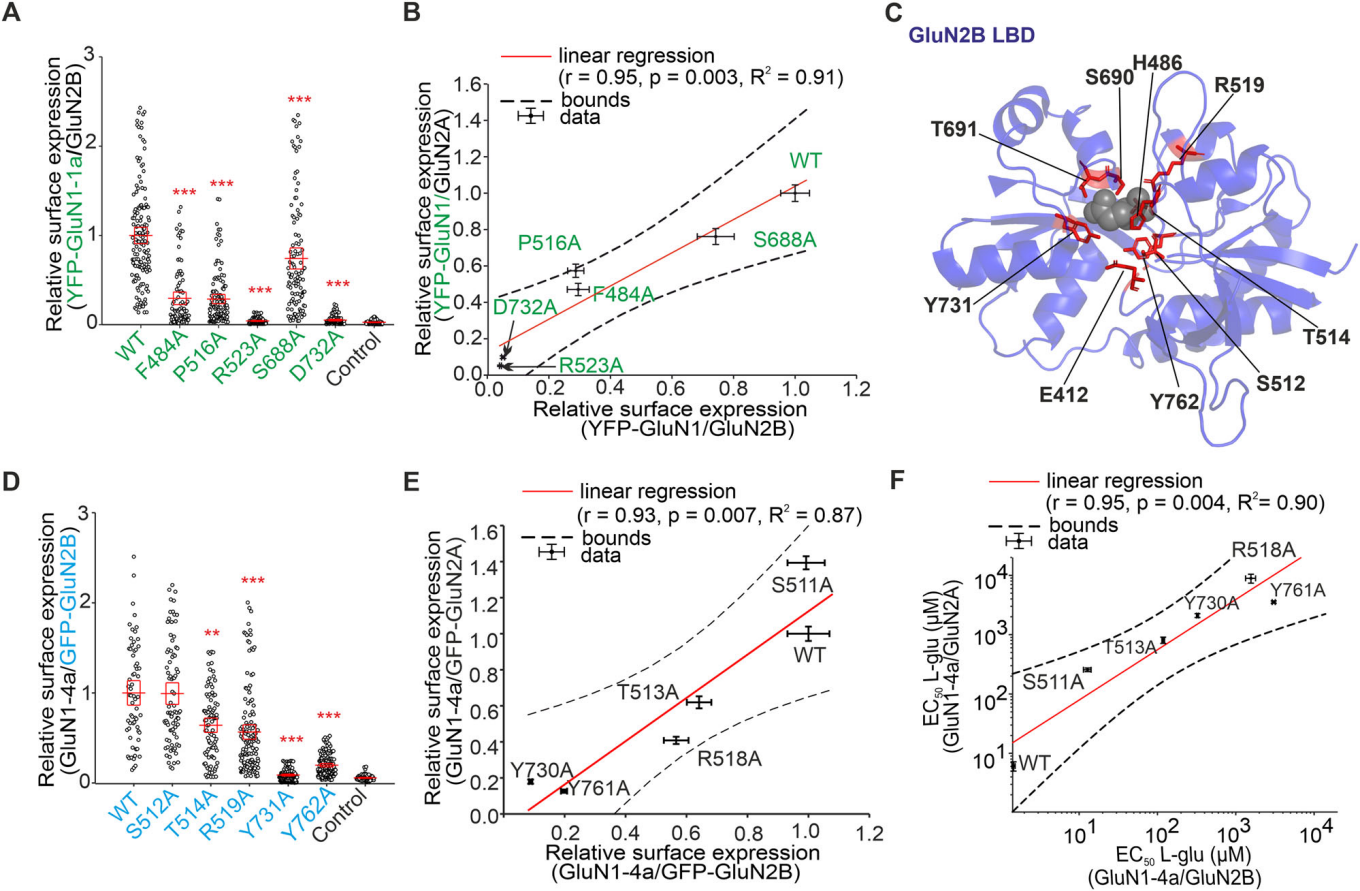
Alanine substitutions in LBDs cause similar changes in surface expression of GluN1/GluN2B receptors as we observed for GluN1/GluN2A receptors. ***A***, Summary of the relative surface expression of NMDARs consisting of either WT or mutated YFP-GluN1-1a subunits coexpressed with the GluN2B subunit, measured using fluorescence microscopy; ***p < 0.001 versus WT, one-way ANOVA. Data points correspond to individual cells (*n* ≥ 84), and the red box plot indicates mean ± SEM. ***B***, Correlation analysis of relative surface expression of YFP-GluN1-1a/GluN2A and YFP-GluN1-1a/GluN2B receptors. ***C***, Structural model of the LBD of the GluN2B subunit labeled with l-glutamate–interacting amino acids (based on PDB ID, 4PE5). ***D***, Summary of the relative surface expression of NMDARs consisting of either WT or mutated GFP-GluN2B subunit coexpressed with the WT GluN1-4a subunit, measured using fluorescence microscopy; *p < 0.050, **p < 0.010, and ***p < 0.001 versus WT; one-way ANOVA. Data points correspond to individual cells (*n* ≥ 64), and the red box plot indicates mean ± SEM. ***E***, Correlation analysis between relative surface expression levels of homologous mutations in GluN2A and GluN2B subunits when coexpressed with the WT GluN1-4a subunit. The plotted points in the graph were labeled according to the amino acid positions of the GluN2A subunit. For normalized concentration–response curves, see Figure S5, and for fitting parameters, see Table 3. ***F***, Correlation analysis between EC_50_ values measured for WT and mutated GluN1/GluN2A and GluN1/GluN2B receptors. The plotted points in the graph were labeled according to the amino acid positions of the GluN2A subunit.

We next asked whether the selected alanine substitutions in LBD of the GluN1 subunit, GluN1-F484A, GluN1-P516A, GluN1-R523A, GluN1-S688A, and GluN1-D732A, examined in more detail in combination with the GluN2A subunit, alter similarly the surface expression of the GluN1/GluN2B receptor. Using immunostaining with an anti-GFP antibody, we observed that the relative surface expression levels of the YFP-GluN1-1a/GluN2B receptors showed a similar order to that of the YFP-GluN1-1a/GluN2A receptors, specifically WT>GluN1-S688A>GluN1-F484A>GluN1-P516A>GluN1-D732A>GluN1-R523A (Fig. 5*A*). After plotting the surface expression values with linear regression, we obtained a very strong positive correlation (*r* = 0.95; *p* = 0.003; *R*^2^ = 0.91) between WT and mutant YFP-GluN1-1a/GluN2A and YFP-GluN1-1a/GluN2B receptors, indicating that the surface expression of both subtypes is similarly regulated by alanine substitutions in LBD of the GluN1 subunit (Fig. 5*B*).

We further investigated whether the surface expression of GluN1/GluN2B receptors is regulated by alanine substitutions in the LBD of the GluN2B subunit, similar to the case of GluN1/GluN2A receptors. The structure of the LBD of the GluN2B subunit with labeled amino acid residues directly interacting with l-glutamate is shown in Figure 5*C*; interestingly, the amino acid residues interacting with l-glutamate are conserved in all GluN2 subunits (Kinarsky et al., 2005). Therefore, we further compared the surface expression of the GluN1-4a/GFP-GluN2B receptors containing the corresponding alanine substitutions, GluN2B-S512A, GluN2B-T514A, GluN2B-R519A, GluN2B-Y731A, and GluN2B-Y761A, which we studied in detail in the case of GluN1/GluN2A receptors. Analysis of the surface fluorescence signals of the GluN1-4a/GFP-GluN2B receptors expressed in the HEK293 cells showed the following order: WT>GluN2B-S512A>GluN2B-T514A>GluN2B-R519A>GluN2B-Y762A>GluN2B-Y731A (Fig. 5*D*), which, except for mutations causing highly deficient surface expression (GluN2B-R519A, GluN2B-Y761A, GluN2B-Y731A), corresponded to the order obtained for homologous mutations in the case of the GluN2A subunit (Fig. 2). This conclusion was confirmed by the very strong positive correlation (*r* = 0.93; *p* = 0.007; *R*^2^ = 0.87) between the relative surface expression of the GluN1/GluN2A and GluN1/GluN2B receptors containing the corresponding alanine substitutions within the LBDs of the GluN2A and GluN2B subunits, respectively (Fig. 5*E*). Our subsequent electrophysiological recordings and concentration-dependent analysis of WT and mutant GluN1-4a/GFP-GluN2B receptors revealed a very strongly positive correlation (*r* = 0.95; *p* = 0.004; *R*^2^ = 0.90) between the EC_50_ values for l-glutamate measured for the GluN1/GluN2A and GluN1/GluN2B receptors (Fig. 5*F*; Fig. S5; Table 3), showing that homologous alanine substitutions in LBDs of the GluN2A and GluN2B subunits have similar effects on the sensitivity of the mutated GluN1/GluN2 receptors to l-glutamate.

**Table 3.** Summary of fitting parameters for steady-state concentration–response curves for l-glutamate measured in HEK293 cells expressing the indicated GluN1/GluN2B receptors

Receptor	EC_50_ (μM)	*I*_max_	*h*	*n*
GluN1-4a/GFP-GluN2B	1.46 ± 0.07	0.98 ± 0.01	1.22 ± 0.07	9
GluN1-4a/GFP-GluN2B-S512A	12.80 ± 1.57	1.01 ± 0.00	1.43 ± 0.06	6
GluN1-4a/GFP-GluN2B-T514A	118.58 ± 4.97	1.06 ± 0.00	1.14 ± 0.03	7
GluN1-4a/GFP-GluN2B-R519A	1,562.84 ± 263.28	1.08 ± 0.03	1.35 ± 0.07	4
GluN1-4a/GFP-GluN2B-T691A	867.10 ± 124.90	1.04 ± 0.01	1.24 ± 0.06	5
GluN1-4a/GFP-GluN2B-Y731A	324.94 ± 9.68	1.03 ± 0.01	1.19 ± 0.03	9
GluN1-4a/GFP-GluN2B-Y762A	3,053.74 ± 155.64	1.24 ± 0.02	1.22 ± 0.05	5

The EC_50_, *I*_max_, and *h* values were obtained by fitting the data from individual cells to Equation 1.

### Pathogenic variants of the specific amino acid residues in LBDs of GluN1 and GluN2A subunits indicate tight regulation of the surface delivery of GluN1/GluN2A receptors

**Figure 6.**
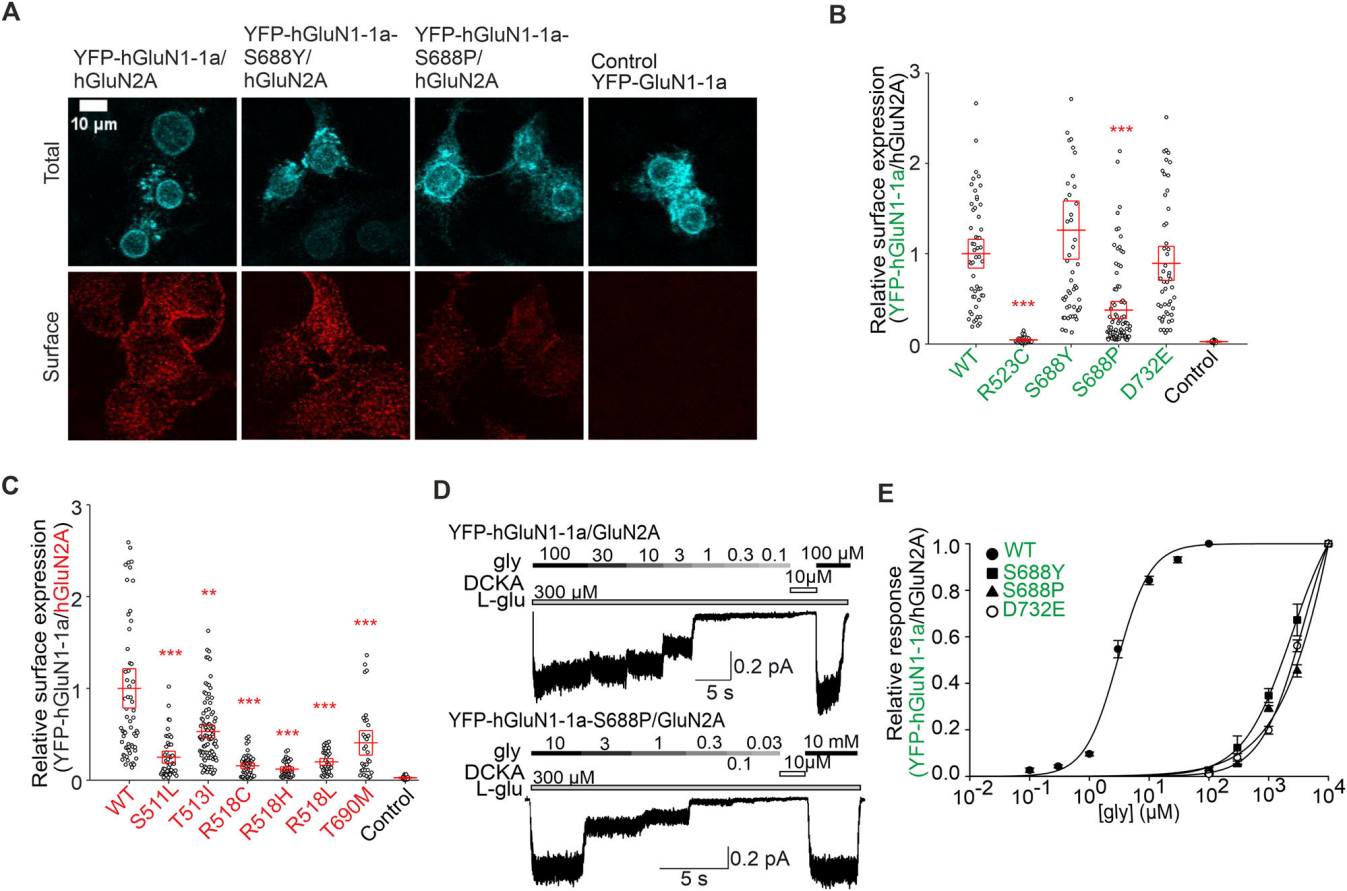
Pathogenic variants in LBDs of both GluN1 and GluN2A subunits affect the surface expression and agonist potency of NMDARs. ***A***, Representative images from HEK293 cells transfected with the WT or mutated YFP-hGluN1-1a subunit either alone or with the hGluN2A subunit. The total and the surface signals (top and bottom row, respectively) of YFP-hGluN1-1a subunits were labeled using an anti-GFP antibody 24 h after the transfection. ***B***, ***C***, Summary of relative surface expression of YFP-hGluN1-1a/hGluN2A receptors containing pathogenic variants within the LBDs of the YFP-hGluN1-1a (***B***) or hGluN2A (***C***) subunits, measured using fluorescence microscopy; **p < 0.010; ***p < 0.001 versus respective WT; one-way ANOVA. Data points correspond to individual cells (*n* ≥ 16), and the red box plot indicates mean ± SEM. ***D***, Representative whole-cell voltage–clamp recordings from HEK293 cells transfected with the indicated NMDAR subunits. Glycine (gly) at the indicated concentrations was applied in the continuous presence of 300 µM l-glutamate (l-glu). ***E***, Normalized concentration–response curves for glycine measured from HEK293 cells expressing WT or mutated YFP-hGluN1-1a/hGluN2A receptors obtained by fitting the data using Equation 1 (see Materials and Methods); for the summary of fitting parameters, see Table 5.

We have searched the “GRIN variants DATABASE” (available at URL https://alf06.uab.es/grindb/home) for pathogenic and potentially pathogenic “missense” variants (hereafter referred to as “pathogenic variants”) of amino acid residues in LBDs involved in the direct interaction of glycine with the GluN1 subunit and l-glutamate with the GluN2A subunit; their list as of January 1, 2022, including relevant information, is provided in Table 4. To examine the effect of the pathogenic variants on the surface expression of GluN1/GluN2A receptors, we chose to combine YFP-hGluN1-1a and hGluN2A subunits, given that we do not have a functional tagged version of the hGluN2A subunit. We labeled the HEK293 cells transfected with WT or mutated YFP-hGluN1-1a/hGluN2A receptors using an anti-GFP antibody (Fig. 6*A*); analysis of microscopical data revealed the following trends of surface expression levels for GluN1 subunit, GluN1-S688Y>WT>GluN1-D732E>GluN1-S688P>GluN1-R523C (Fig. 6*B*), and for GluN2A subunit, WT>GluN2A-T513I>GluN2A-T690M>GluN2A-S511L>GluN2A-R518L>GluN2A-R518C>GluN2A-R518H (Fig. 6*C*). We then performed the same electrophysiological approach described above using the HEK293 cells expressing WT and mutant YFP-hGluN1-1a/hGluN2A receptors, and we analyzed concentration–response curves for glycine (for pathogenic variants in the GluN1 subunit) and l-glutamate (for pathogenic variants in the GluN2A subunit; Fig. 6*D*). Concerning the pathogenic variants in the GluN1 subunit, we did not detect any current responses in HEK293 cells transfected with the YFP-hGluN1-1a-R523C/hGluN2A receptor. In contrast, we observed a profound 3,188-fold increase in EC_50_ values for glycine with YFP-hGluN1-1a-S688P/hGluN2A, a 944-fold increase for YFP-hGluN1-1a-S688Y/hGluN2A, and a 1,457-fold increase for YFP-hGluN1-1a-D732E/hGluN2A receptors (Fig. 6*E*). Concerning the pathogenic variants in the GluN2A subunit, we did not detect any current responses of YFP-hGluN1-1a/hGluN2A-S511L, YFP-hGluN1-1a/hGluN2A-R518H, YFP-hGluN1-1a/hGluN2A-R518L, and YFP-hGluN1-1a/hGluN2A-T690M receptors even in the presence of 10 mM l-glutamate. In the case of the YFP-hGluN1-1a/hGluN2A-T513I and YFP-hGluN1-1a/hGluN2A-R518C receptors, we observed current responses only in the presence of 3 and 10 mM l-glutamate, and we did not proceed with the concentration–response analysis. These experiments showed that pathogenic variants in LBDs can have a prominent impact, distinct from the corresponding alanine substitutions, on regulating the surface expression of GluN1/GluN2A receptors.

**Table 4.** Pathogenic and potential pathogenic variants in LBDs of GluN1 and GluN2A investigated in this study

Variant	Gene	Genotype	Phenotype	References
GluN1-R523C	*GRIN1*	c.1567C > T	DA	(Kalbaugh et al., 2004); ClinVar ID: 1308763
GluN1-S688Y	*GRIN1*	c.2063C > A	Early infantile encephalopathy, intellectual disability	(Zehavi et al., 2017; Skrenkova et al., 2020; Chen et al., 2023); ClinVar ID: 981272
GluN1-S688P	*GRIN1*	c.2062T > C	DA	ClinVar ID: 429761
GluN1-D732E	*GRIN1*	-	DA	(Santos-Gómez et al., 2021)
GluN2A-S511L	*GRIN2A*	c.1532C > T	Landau–Kleffner syndrome	ClinVar ID: 80290
GluN2A-T513I	*GRIN2A*	c.1538C > T	Landau–Kleffner syndrome	ClinVar ID: 424392
GluN2A-R518C	*GRIN2A*	c.1552C > T	Landau–Kleffner syndrome	(Strehlow et al., 2019); ClinVar ID: 205687
GluN2A-R518H	*GRIN2A*	c.1553G > A	Landau–Kleffner syndrome	(Lesca et al., 2013; Conroy et al., 2014; Swanger et al., 2016; Strehlow et al., 2019); ClinVar ID: 88732
GluN2A-R518L	*GRIN2A*	c.1553G > T	Landau–Kleffner syndrome	ClinVar ID: 567708
GluN2A-T690M	*GRIN2A*	c.2069C > T	Inborn genetic diseases, Landau–Kleffner syndrome, neurodevelopmental disorder	(Nykamp et al., 2017; Fernández-Marmiesse et al., 2019); ClinVar ID: 444362

The variants were selected from the open-source GRIN database (https://alf06.uab.es/grindb/home). The information provided is current as of January 1, 2022. Variants marked as “DA” denote disease-associated or potential pathogenic variants.

### The mutant cycles of the specific amino acid residues in LBDs of GluN1 and GluN2A subunits indicate tight regulation of the surface expression of GluN1/GluN2A receptors

**Figure 7.**
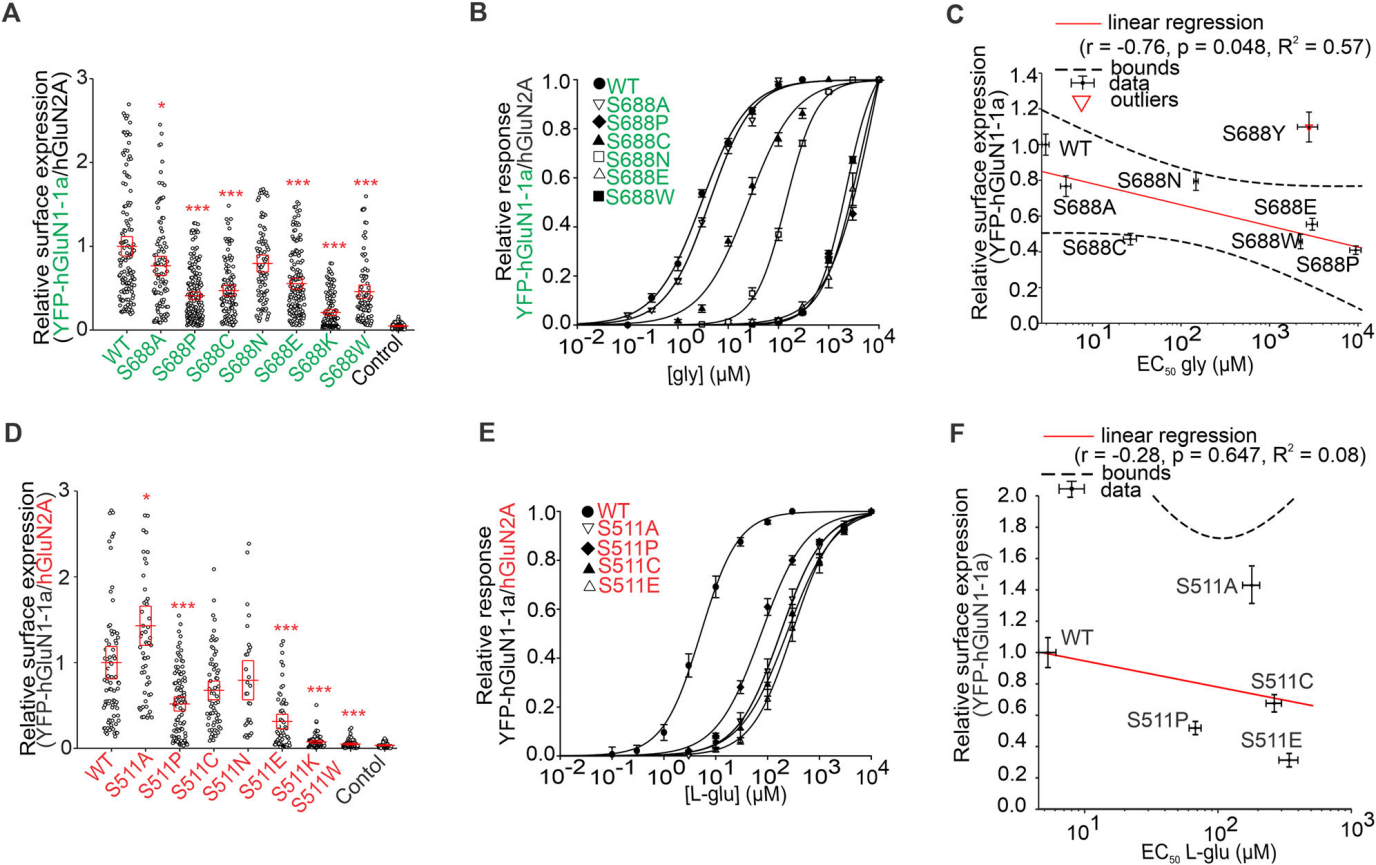
Microscopical and electrophysiological characterization of the mutant cycles of the GluN1-S688 and GluN2A-S511 residues. ***A***, Summary of the relative surface expression of NMDARs containing the indicated mutations of the GluN1-S688 residue, measured using fluorescence microscopy; *p < 0.050 and ***p < 0.001 versus WT; one-way ANOVA. Data points correspond to individual cells (*n* ≥ 82), and the red box plot indicates mean ± SEM. ***B***, Normalized concentration–response curves for glycine (gly) measured from HEK293 cells expressing WT and mutant NMDARs were obtained by fitting the data using Equation 1 (see Materials and Methods); for the summary of fitting parameters, see Table 5. ***C***, Correlation analysis of surface expression and EC_50_ values for glycine for WT and mutated YFP-hGluN1-1a/hGluN2A receptors containing the S688 residue mutant cycle variants, fitted by linear regression. For *K*_d_ value estimations, see Figure S6; for the correlation analysis of EC_50_ and *K*_d_ values for glycine, see Figure S7. ***D***, Summary of the relative surface expression of NMDARs containing the indicated mutations, measured using fluorescence microscopy; *p < 0.050 and ***p < 0.001 versus WT; one-way ANOVA. Data points correspond to individual cells (*n* ≥ 29), and the red box plot indicates mean ± SEM. ***E***, Normalized concentration–response curves for l-glutamate (l-glu) measured from HEK293 cells expressing NMDARs containing mutations of the GluN2A-S511 residue obtained by fitting the data using Equation 1 (see Materials and Methods); for the summary of fitting parameters, see Table 5. ***F***, Correlation analysis of relative surface expression values and EC_50_ values for l-glutamate for WT and mutated YFP-hGluN1-1a/hGluN2A receptors fitted by linear regression. For *K*_d_ value estimations, see Figure S8; for the correlation analysis of EC_50_ and *K_d_* values for l-glutamate, see Figure S9.

Interestingly, the surface expression levels determined for YFP-hGluN1-1a/hGluN2A receptors containing pathogenic variants were profoundly different compared with the corresponding alanine substitutions for GluN1-S688 (∼76% for the GluN1-S688A mutation compared with ∼126% for the hGluN1-S688Y or ∼38% for the hGluN1-S688P variants; ∼10% for the GluN1-D732A mutation vs ∼89% for the hGluN1-D732E variant; ∼139% for the GluN2A-S511A mutation vs ∼25% for the hGluN2A-S511L variant; ∼81% for the GluN2A-T690A mutation vs ∼41% for the hGluN2A-T690M variant). Considering the practical scope of subsequent experimental and in silico analyses, we designed a series of mutant cycles targeting the GluN1-S688 and GluN2A-S511 residues. Specifically, we introduced substitutions in the YFP-hGluN1-1a and hGluN2A subunits for alanine (A; small uncharged), cysteine (C; nucleophilic), proline (P; hydrophobic), tryptophan (W; aromatic), glutamic acid (E; acidic), asparagine (N; amide), and lysine (K; basic). Regarding the mutant cycle of the GluN1-S688 residue, our microscopical analysis showed the following trend for the surface expression levels of YFP-hGluN1-1a/hGluN2A receptors expressed in HEK293 cells: WT>GluN1-S688N>GluN1-S688A>GluN1-S688E>GluN1-S688C>GluN1-S688W>GluN1-S688P>GluN1-S688K (Fig. 7*A*). Our electrophysiological measurements showed that all YFP-hGluN1-1a/hGluN2A receptors containing the mutant GluN1-S688 residue cycle produced sufficient amplitudes of the current responses to obtain the EC_50_ values for glycine (Fig. 7*B*; Table 5). Subsequent analysis revealed a moderate negative correlation (*r* = −0.76), explaining 76% of the variance (*R*^2^ = 0.57) between the surface expression levels and EC_50_ values for glycine at WT and mutant YFP-hGluN1-1a/hGluN2A receptors containing the GluN1-S688 residue mutant cycle; this correlation was statistically significant (*p* = 0.048; Fig. 7*C*). To corroborate our measured EC_50_ values for glycine, we estimated the *K*_d_ values for glycine at WT and mutant YFP-hGluN1-1a/hGluN2A receptors by assessing *τ*_on_ and *τ*_off_ values from the current responses elicited by different concentrations of glycine in the presence of 100 µM l-glutamate (Fig. S6; Text S1). Correlation analysis of the EC_50_ and *K*_d_ values for WT and mutant YFP-hGluN1-1a/hGluN2A receptors containing the GluN1-S688 residue mutant cycle showed a very strong correlation (*r* = 1.00), with a *p* value of 0.00 and *R*^2^ = 1.00 (Fig. S7; the fitting parameters in Table S1).

**Table 5.** Summary of fitting parameters for steady-state concentration–response curves for glycine and l-glutamate measured in HEK293 cells expressing the human versions of the GluN1/GluN2A receptors carrying the indicated pathogenic variants and the mutant cycle substitutions

Receptor	Agonist	EC_50_ (μM)	*I*_max_	*h*	*n*
YFP-hGluN1-1a/hGluN2A	Glycine	2.96 ± 0.29	1.01 ± 0.02	0.96 ± 0.08	6
YFP-hGluN1-1a-R523C/hGluN2A	Glycine	NR	-	-	10
YFP-hGluN1-1a-S688A/hGluN2A	Glycine	5.02 ± 0.75	1.04 ± 0.1	0.96 ± 0.04	5
YFP-hGluN1-1a-S688Y/hGluN2A	Glycine	2,795.62 ± 787.88	1.26 ± 0.09	1.12 ± 0.11	5
YFP-hGluN1-1a-S688P/hGluN2A	Glycine	9,435.59 ± 1,533.21	1.93 ± 0.15	0.96 (Fixed)	5
YFP-hGluN1-1a-S688C/hGluN2A	Glycine	26.88 ± 5.07	0.99 ± 0.02	0.97 ± 0.07	5
YFP-hGluN1-1a-S688N/hGluN2A	Glycine	147.66 ± 9.17	1.02 ± 0.00	1.38 ± 0.08	7
YFP-hGluN1-1a-S688E/hGluN2A	Glycine	3,044.67 ± 496.54	1.18 ± 0.04	1.49 ± 0.15	4
YFP-hGluN1-1a-S688K/hGluN2A	Glycine	NA^a^	-	-	>60
YFP-hGluN1-1a-S688W/hGluN2A	Glycine	2,249.31 ± 105.92	1.11 ± 0.01	1.49 ± 0.04	8
YFP-hGluN1-1a/hGluN2A	l-Glutamate	5.31 ± 0.88	0.99 ± 0.01	1.28 ± 0.04	5
YFP-hGluN1-1a/hGluN2A-S511L	l-Glutamate	NR	-	-	11
YFP-hGluN1-1a/hGluN2A-S511A	l-Glutamate	179.45 ± 29.45	1.00 ± 0.00	1.05 ± 0.02	5
YFP-hGluN1-1a/hGluN2A-S511P	l-Glutamate	67.71 ± 8.08	0.96 ± 0.00	1.14 ± 0.04	4
YFP-hGluN1-1a/hGluN2A-S511C	l-Glutamate	264.49 ± 38.94	1.00 ± 0.01	1.00 ± 0.04	5
YFP-hGluN1-1a/hGluN2A-S511N	l-Glutamate	NR	-	-	8
YFP-hGluN1-1a/hGluN2A-S511E	l-Glutamate	342.32 ± 61.05	1.02 ± 0.01	1.13 ± 0.06	6
YFP-hGluN1-1a/hGluN2A-S511K	l-Glutamate	NR	-	-	8
YFP-hGluN1-1a/hGluN2A-S511W	l-Glutamate	NR	-	-	5

The EC_50_, *I*_max_, and *h* values were obtained by fitting the data from individual cells to Equation 1. NR indicates “not responding,” and NA indicates “not analyzed.”

a In initial experiments with the GluN1-S688K/GluN2A receptor, we observed current responses in the range of 466–877 pA (*n* = 6), but we could not later replicate these results when validating our electrophysiological data during the revisions. For corresponding *K*_d_ values and fitting parameters, see Table S1.

Regarding the mutant cycle of the GluN2A-S511 residue, we observed the following trend in the surface expression of YFP-hGluN1-1a/hGluN2A receptors in HEK293 cells: GluN2A-S511A>WT>GluN2A-S511N>GluN2A-S511C>GluN2A-S511P>GluN2A-S511E>GluN2A-S511K>GluN2A-S511W (Fig. 7*D*). Our electrophysiological measurements from HEK293 cells expressing YFP-hGluN1-1a/hGluN2A receptors containing GluN2A-S511N, GluN2A-S511K, and GluN2A-S511W mutations showed no current responses; therefore, we calculated EC_50_ values for l-glutamate for the remaining mutant YFP-hGluN1-1a/hGluN2A receptors (Fig. 7*E*; Table 5). Using linear regression, we did not observe a correlation (*r* = −0.28; *p* = 0.647; *R*^2^ = 0.08) between the relative surface expression levels and the EC_50_ values for l-glutamate for the YFP-hGluN1-1a/hGluN2A receptors with the mutant cycle of the GluN2A-S511 residue (Fig. 7*F*). Correlation analysis of the EC_50_ and *K*_d_ values for WT and mutant YFP-hGluN1-1a/hGluN2A receptors containing the GluN1-S511 residue mutant cycle showed a very strong correlation (*r* = 1.00), with a *p* value of 0.00 and *R*^2^ = 1.00 (the representative current responses shown in Fig. S8; the correlation analysis in Fig. S9; the fitting parameters in Table S1), corroborating our measurements of the EC_50_ values for l-glutamate.

**Figure 8.**
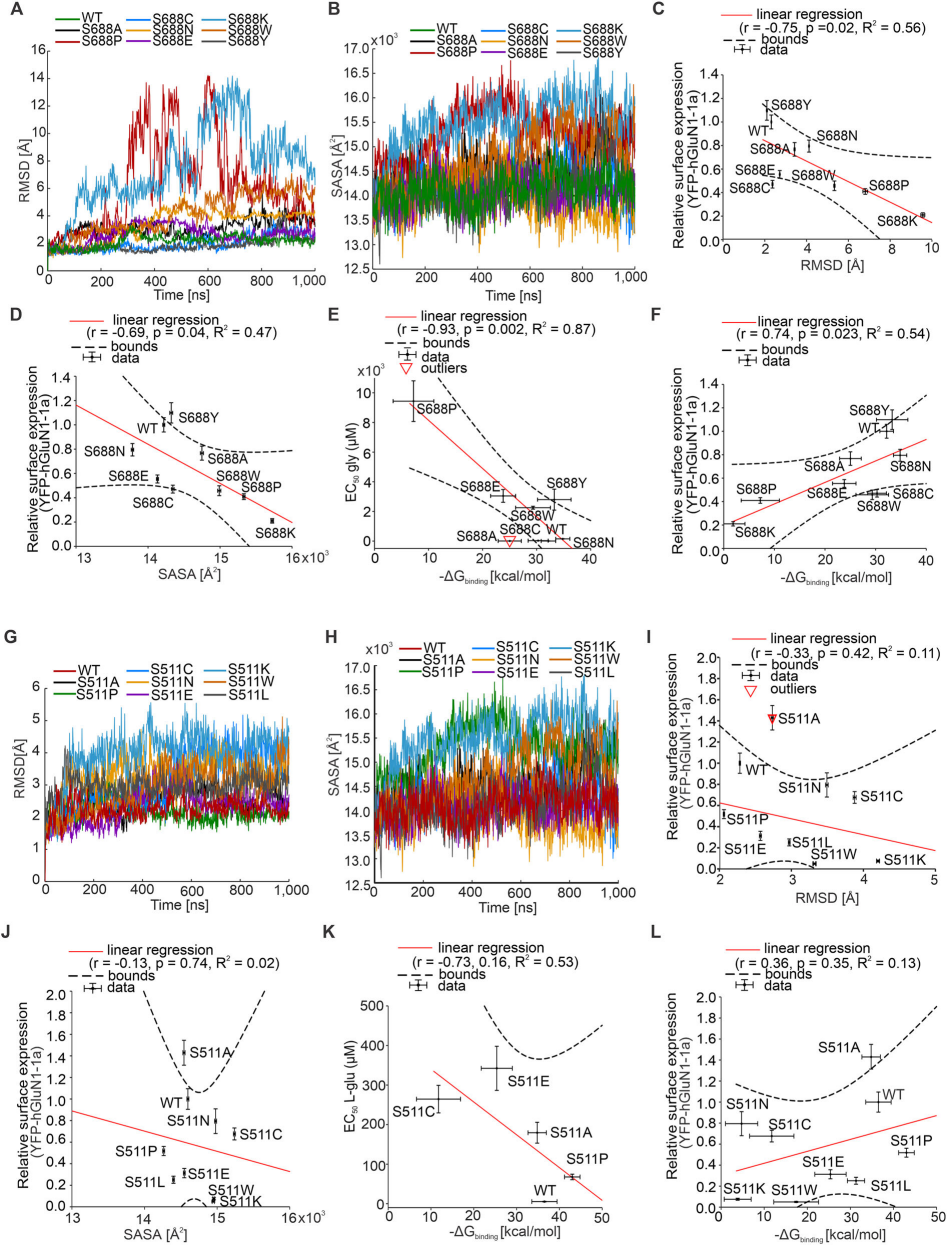
In silico analysis of the mutant cycles of the GluN1-S688 and GluN2A-S511 residues. ***A***, ***G***, The RMSD values were derived from 1-µs-long atomistic simulations of the glycine (***A***) or l-glutamate (***G***) heavy atoms. These values were calculated by least squares fitting the structures to the backbone of the LBDs of either WT GluN1 or mutated GluN1 subunits at S688 residue position (***A***) and WT GluN2A or mutated GluN2A subunits at S511 residue position (***G***; PDB ID, 5KCJ). For RMSF values, see Figures S10 and S11. ***B***, ***H***, The SASA values were derived from 1-µs-long atomistic simulations of the glycine (***B***) or l-glutamate (***H***) heavy atoms. These values were calculated by least squares fitting the structures to the backbone of the LBDs of either WT GluN1 or mutated GluN1 subunits at S688 residue position (***B***) and WT GluN2A or mutated GluN2A subunits at S511 residue position (***H***; PDB, 5KCJ). ***C***, ***I***, Correlation analysis between relative surface expression and RMSD values for the mutant cycles of the GluN1-S688 (***C***) or GluN2A-S511 (***I***) residues. The data were fitted by linear regression. For the summary of RMSD values, see Table 6. ***D***, ***J***, Correlation analysis between relative surface expression and SASA values for the mutant cycles of the GluN1-S688 (***D***) or GluN2A-S511 (***J***) residues. The data were fitted by linear regression. For the summary of SASA values, see Table 6. ***E***, ***K***, Correlation analysis between EC_50_ and Δ*G*_binding_ values for glycine for the mutant cycle of the GluN1-S688 residue (***E***) or l-glutamate for the mutant cycle of the GluN2A-S511 residue (***K***). The data were fitted by linear regression. For the summary of Δ*G*_binding_ values, see Table 6. ***F***, ***L***, Correlation analysis between relative surface expression and Δ*G*_binding_ values for glycine for the mutant cycle of the GluN1-S688 residue (***F***) or l-glutamate for the mutant cycle of the GluN2A-S511 residue (***L***). The data were fitted by linear regression. For the summary of EC_50_ values, see Table 5; for Δ*G*_binding_ values, see Table 6.

To explore the impact of the mutant cycles of the GluN1-S688 and GluN2A-S511 residues on the structural stability of LBDs and their propensity to change the ligand-binding sites locally, we systematically modeled and simulated the individual mutations into the LBDs of GluN1 and GluN2A subunits. The global stability of LBDs of GluN1 and GluN2A subunits, in both WT and mutated forms, was evaluated using a comprehensive set of structural analyses, including the RMSD of protein backbone residues (Fig. 8*A*,*G*), the RMSF of individual residues (Figs. S10, S11), and the SASA (Fig. 8*B*,*H*), derived from 1-µs-long atomistic simulations. The RMSD value for the WT LBD of the GluN1 subunit was 2.31 ± 0.01 Å, indicating a stable structure closely aligned with the crystal form. The RMSD values for the LBDs of the mutated GluN1 subunit exhibited relatively large differences among WT and mutated LBDs of the GluN1 subunit (Fig. 8*A*). Our analysis revealed a strong negative correlation (*r* = −0.75), explaining 75% of the variance (*R*^2^ = 0.56) between the relative surface expression levels and RMSD values for the WT and the GluN1-S688 residue mutant cycle; this correlation was statistically significant (*p* = 0.02; Fig. 8*C*). The LBD structures containing the two most extreme mutations, GluN1-S688P and GluN1-S688K, showed RMSD values of 6.79 ± 0.11 Å and 9.55 ± 0.10 Å, respectively, indicating substantial deviations from the crystal structure. In addition, these mutations led to the adoption of more extended LBD conformations that effectively opened the glycine access/egress pathway, contrasting with the more compact conformation observed in the crystal structure (Table 6). Next, our calculations revealed the SASA value of 14,217.90 ± 14.26 Å^2^ for WT LBD of the GluN1 subunit; the subsequent analysis showed a moderately strong negative correlation (*r* = −0.69), explaining 69% of the variance (*R*^2^ = 0.47) between the relative surface expression levels and SASA values for the WT and the GluN1-S688 residue mutant cycle; this correlation was statistically significant (*p* = 0.04; Fig. 8*D*). Consistently with our findings described above, we revealed an increase in SASA values to 15,330.34 ± 16.80 Å^2^ and 15,723.66 ± 17.28 Å^2^ for GluN1-S688P and GluN1-S688K mutations, respectively, supporting the hypothesis that both mutations transition the LBD to more extended conformation, underscoring the shift toward a less compact and more solvent-exposed structure. Furthermore, the LBDs containing GluN1-S688P and GluN1-S688K mutations exhibited profound RMSF deviations compared with the WT LBD, and the global structural alterations in the LBDs induced by GluN1-S688P and GluN1-S688K mutations led to the dissociation of glycine from the ligand-binding site (Table 6). Finally, by conducting MM-PBSA calculations, we obtained an Δ*G*_binding_ value of −32.18 ± 1.35 kcal/mol for the WT LBD of the GluN1 subunit, consistent with the previous calculation (Chen et al., 2023). The calculated Δ*G*_binding_ values for the WT and mutant LBDs of the GluN1 subunit containing the GluN1-S688 residue mutant cycle exhibited a very strong negative correlation (*r* = −0.93), explaining 93% of the variance (*R*^2^ = 0.87) when compared with the experimentally measured EC_50_ values for glycine; this correlation was statistically significant (*p* = 0.002; Fig. 8*E*). In addition, we observed a strong correlation (*r* = 0.74), explaining 74% of the variance (*R*^2^ = 0.54) between the relative surface expression and the Δ*G*_binding_ values for glycine for the GluN1-S688 residue mutant cycle; this correlation was statistically significant (*p* = 0.023; Fig. 8*F*). Consistently with the above-described findings, we observed Δ*G*_binding_ values of −7.21 ± 3.77 kcal/mol and −1.84 ± 2.35 kcal/mol for the LBDs containing the GluN1-S688P and GluN1-S688K mutations, respectively, indicating that both mutations profoundly destabilize the glycine-binding site (Table 6).

**Table 6.** Summary of RMSD, SASA, and Δ*G*_binding_ values for LBDs of the GluN1 subunit for glycine and the GluN2A subunit for l-glutamate obtained by MD simulations

LBD	Agonist	RMSD [Å]	SASA [Å^2^]	Δ*G*_binding_ [kcal/mol]
hGluN1	Glycine	2.31 ± 0.01	14,217.90 ± 14.26	−32.18 ± 1.35
hGluN1-S688A	Glycine	3.42 ± 0.03	14,747.81 ± 21.12	−25.02 ± 2.14
hGluN1-S688Y	Glycine	2.10 ± 0.02	14,321.65 ± 20.05	−33.27 ± 3.10
hGluN1-S688P	Glycine	6.79 ± 0.11	15,330.34 ± 16.80	−7.21 ± 3.77
hGluN1-S688C	Glycine	2.36 ± 0.02	14,343.69 ± 18.10	−30.27 ± 1.86
hGluN1-S688N	Glycine	4.11 ± 0.01	13,783.14 ± 16.53	−34.84 ± 1.30
hGluN1-S688E	Glycine	2.71 ± 0.02	14,130.32 ± 13.76	−23.80 ± 2.31
hGluN1-S688K	Glycine	9.55 ± 0.10	15,723.66 ± 17.28	−1.84 ± 2.35
hGluN1-S688W	Glycine	5.32 ± 0.03	14,988.77 ± 21.89	−29.33 ± 3.22
hGluN2A	l-Glutamate	2.28 ± 0.01	14,596.85 ± 13.91	−36.50 ± 2.95
hGluN2A-S511L	l-Glutamate	2.97 ± 0.01	14,398.11 ± 13.23	−31.31 ± 2.00
hGluN2A-S511A	l-Glutamate	2.74 ± 0.02	14,542.83 ± 15.59	−34.81 ± 2.15
hGluN2A-S511P	l-Glutamate	2.06 ± 0.01	14,263.28 ± 14.01	−43.01 ± 1.81
hGluN2A-S511C	l-Glutamate	3.88 ± 0.02	15,237.64 ± 13.77	−11.76 ± 5.14
hGluN2A-S511N	l-Glutamate	3.49 ± 0.02	14,978.40 ± 14.27	−4.75 ± 3.75
hGluN2A-S511E	l-Glutamate	2.57 ± 0.02	14,547.50 ± 12.73	−25.37 ± 3.64
hGluN2A-S511K	l-Glutamate	4.20 ± 0.02	14,954.19 ± 13.85	−3.85 ± 3.13
hGluN2A-S511W	l-Glutamate	3.32 ± 0.02	14,946.35 ± 15.09	−17.42 ± 5.21

RMSD and SASA values were calculated from the last 500 ns of the 1-µs-long simulation for each LBD structure; see Materials and Methods section for more details.

The crystal structure was largely conserved for the WT LBD of the GluN2A subunit, as indicated by an overall RMSD value of 2.28 ± 0.01 Å. The RMSD values for the GluN2A-S511 residue mutant cycle did not exceed 4.2 Å, with the highest deviation observed for the GluN2A-S511K mutation (Table 6). This indicates that the overall fold of LBDs of the GluN2A subunit remained stable despite the GluN2A-S511 residue mutations. Our analysis revealed no correlation (*r* = −0.33; *R*^2^ = 0.11; *p* = 0.42) between the relative surface expression levels and RMSD values for the WT and mutant LBDs containing the GluN2A-S511 residue mutant cycle (Fig. 8*I*; Table 6). The SASA values for the LBDs containing the mutant cycle of the GluN2A-S511 residue also remained relatively consistent, supporting the notion of a preserved structural integrity of the LBD of the GluN2A subunit containing different mutations. Our analysis showed no correlation (*r* = −0.13; *R*^2^ = 0.02; *p* = 0.74) between the relative surface expression levels and SASA values for the WT and mutant LBDs containing the GluN2A-S511 residue mutant cycle (Fig. 8*J*). Furthermore, the LBDs of the GluN2A subunit containing the GluN2A-S511 residue mutant cycle exhibited only slight RMSF deviations compared with the WT LBD (Fig. S11). Finally, we calculated the Δ*G*_binding_ value of −36.50 ± 2.95 kcal/mol for l-glutamate binding into the WT LBDs of the GluN2A subunit and the GluN2A-S511 residue mutant cycle. We also observed relatively strong l-glutamate interaction with the LBDs containing the GluN2A-S511P (−43.01 ± 1.81 kcal/mol), GluN2A-S511A (−34.81 ± 2.15 kcal/mol), and GluN2A-S511L (31.31 ± 2.00 kcal/mol) mutations. The least favorable l-glutamate binding was observed for the S511E mutation (−25.37 ± 3.64 kcal/mol); likely, the negative charge introduced by the S511E mutation destabilized the interaction with the negatively charged l-glutamate. Interestingly, we observed no dissociation of l-glutamate for the above-tested mutated LBDs of the GluN2A subunit. In the case of S511W, S511C, S511N, and S511K mutations, spontaneous dissociation of l-glutamate was observed, resulting in distinctly lower Δ*G*_binding_ values of −17.42 ± 5.21 kcal/mol, −11.76 ± 5.14 kcal/mol, −4.75 ± 3.75 kcal/mol, and −3.85 ± 3.13 kcal/mol, respectively (Table 6). Unexpectedly, in none of these cases where the l-glutamate spontaneously dissociated from the LBD, we did not observe any structural changes underlying this process. This indicates that the less compact structure of the LBD of the GluN2A subunit enables the l-glutamate to bind and unbind more readily than we observed during simulations of glycine interaction with the LBD of the GluN1 subunit. The calculated Δ*G*_binding_ values for the WT and mutant LBDs of the GluN2A subunit containing the GluN2A-S511 residue mutant cycle exhibited no correlation (*r* = −0.73; *p* = 0.16; *R*^2^ = 0.53) compared with the measured EC_50_ values for l-glutamate (Fig. 8*K*). The lack of correlation between calculated Δ*G*_binding_ and the experimentally measured EC_50_ values for l-glutamate could be explained by the more open conformation of the LBD of the GluN2A subunit with the exposed ligand-binding site; this structural characteristic allows spontaneous ligand dissociation without global structural reengagements. We also cannot exclude the effect of the cooperativity of the LBDs in the NMDAR heterotetramer, which may lead to no correlation between EC_50_ and Δ*G*_binding_ values. In addition, we observed no correlation (*r* = 0.36; *R*^2^ = 0.13; *p* = 0.35) between the relative surface expression and the Δ*G*_binding_ values for l-glutamate for the GluN2A-S511 residue mutant cycle (Fig. 8*L*). These analyses revealed distinct behaviors between LBDs of GluN1 and GluN2A subunits and highlighted profound structural differences among individual systems of the LBDs containing the GluN1-S688 and GluN2A-S511 residue mutant cycles.

**Figure 9.**
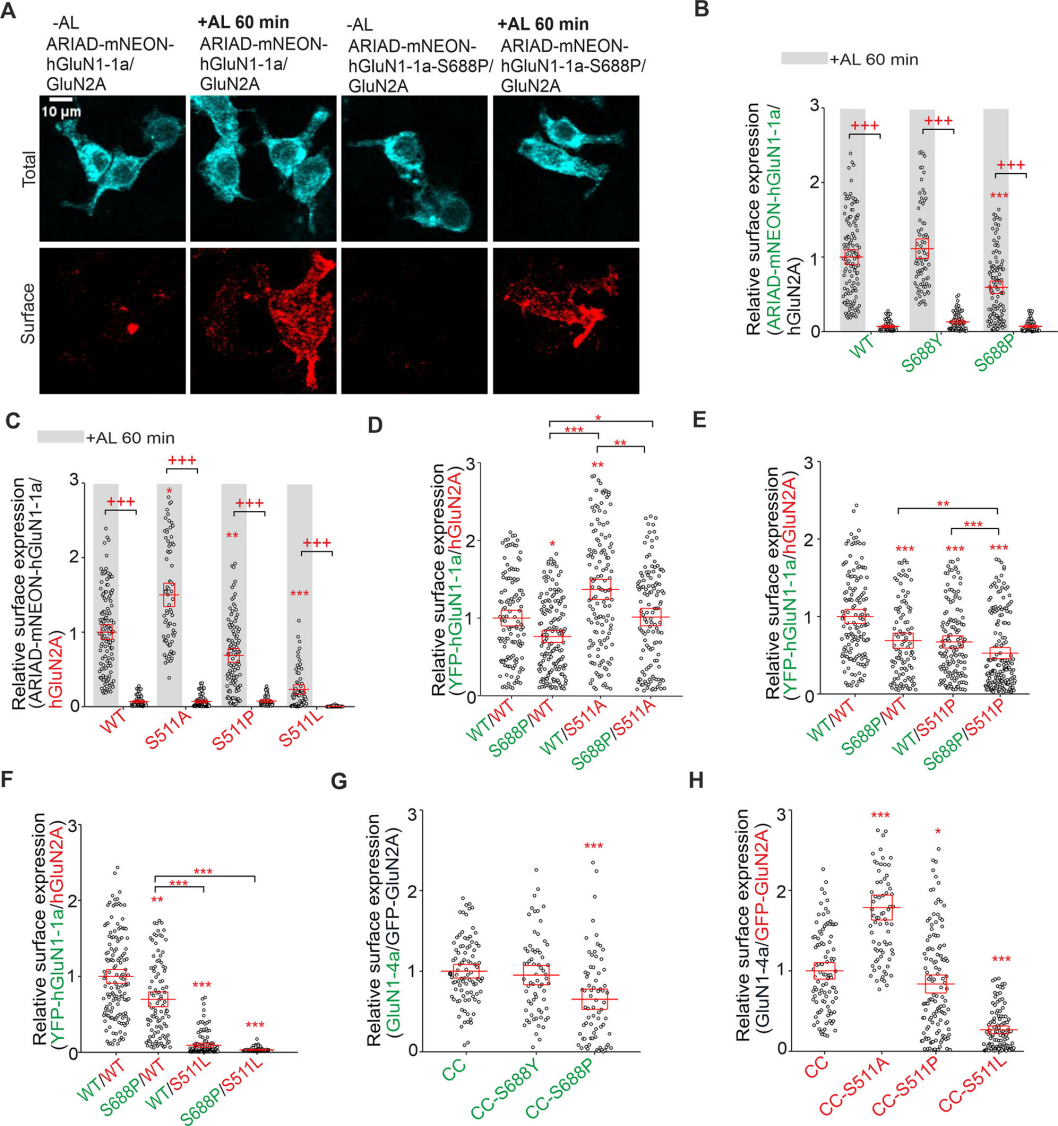
The role of the pathogenic variants and other mutations in LBDs of both GluN1 and GluN2A subunits in the regulation of the surface expression of NMDARs. ***A***, Representative images of HEK293 cells expressing ARIAD-mNEON-hGluN1-1a construct either alone or with the GluN2A subunit at 0 min (without AL) and 60 min after adding AL. The total and surface signals (top and bottom row, respectively) of ARIAD-mNEON-hGluN1-1a subunits were labeled using an anti-mNEONGreen antibody 24 h after the transfection. ***B***, ***C***, Summary of relative surface expression of NMDARs containing WT or mutated ARIAD-mNEON-hGluN1-1a subunits coexpressed together with WT hGluN2A subunit (***B***) or WT ARIAD-mNEON-hGluN1-1a subunit coexpressed with WT or mutated hGluN2A subunits (***C***) measured in the absence or presence (60 min) of AL, using fluorescence microscopy; *p < 0.05, **p < 0.010, and ***p < 0.001 for differences between ARIAD-mNEON-hGluN1-1a/GluN2A receptor and mutated NMDARs in presence of AL; +++*p* < 0.001 for differences between the absence and presence of AL; two-way ANOVA. Data points correspond to individual cells (*n* ≥ 54), and the red box plot indicates mean ± SEM. ***D–F***, Summary of the relative surface expression of NMDARs containing mutations within LBDs of YFP-hGluN1-1a and hGluN2A subunits, measured using fluorescence microscopy; **p < 0.01 and ***p < 0.001 versus WT; one-way ANOVA. Data points correspond to individual cells (*n* ≥ 81), and the red box plot indicates mean ± SEM. ***G***, ***H***, Summary of relative surface expression for NMDARs composed of GluN1-4a and GFP-GluN2A subunits with the closed-cleft (CC) conformation of LBDs in combination with selected substitutions, measured using fluorescence microscopy; *p < 0.05 and ***p < 0.001 versus WT; one-way ANOVA. Data points correspond to individual cells (*n* ≥ 67), and the red box plot indicates mean ± SEM.

In the next phase, we carried out a detailed investigation into the role of specific mutations in the early trafficking of NMDARs, focusing on pathogenic variants including GluN1-S688Y (which did not affect relative surface expression), GluN1-S688P (which decreased relative surface expression by ∼62%; both mutations shown in Fig. 6), and GluN2A-S511L (which reduced relative surface expression to ∼25%), along with two additional mutations, GluN2A-S511A (the only mutation that increased relative surface expression) and GluN2A-S511P (which reduced relative surface expression by ∼48%; all mutations shown in Figs. 2, 6). First, we created the WT ARIAD-hGluN1-1a construct and introduced the hGluN1-S688Y and hGluN1-S688P variants. Cotransfection of HEK293 cells with the WT ARIAD-hGluN1-1a construct and the WT hGluN2A subunit resulted in robust surface expression of the WT mNEON-hGluN1-1a/hGluN2A receptor 60 min after addition of AL but not under control conditions (0 min; Fig. 9*A*,*B*). In line with our data above, the hGluN1-S688Y variant did not affect the relative surface expression of mNEON-hGluN1-1a/hGluN2A receptor measured 60 min after AL addition, while the GluN1-S688P variant reduced the relative surface expression by ∼41% (Fig. 9*A*,*B*). Additionally, coexpressing the WT ARIAD-hGluN1-1a construct with mutated hGluN2A subunits led to either an increase (∼50% for the hGluN2A-S511A mutation) or a decrease (∼31% for hGluN2A-S511P and ∼87% for hGluN2A-S511L variants) of the relative surface expression of mNEON-hGluN1-1a/hGluN2A receptors 60 min after AL addition (Fig. 9*C*). Under control conditions (0 min), essentially no mNEON-hGluN1/hGluN2A receptors were detected on the cell surface (Fig. 9*C*). Thus, our data suggest that the observed changes in surface expression of hGluN1/hGluN2A receptors with the studied mutations, including pathogenic variants in the LBDs, are caused by altered early trafficking in the HEK293 cells.

We then investigated whether the presence of the pathogenic variants and other selected mutations in the LBDs of hGluN1 and hGluN2A subunits is sensed independently by control mechanisms. We cotransfected HEK293 cells with a combination of WT and mutant YFP-hGluN1-1a (containing the S688P variant; the S688Y variant was not assessed as it does not alter the surface delivery of the hGluN1/hGluN2A receptors) and hGluN2A (containing hGluN2A-S511A, hGluN2A-S511P, and hGluN2A-S511L mutations) subunits. Our analysis revealed that in all cases, the combination of two mutated hGluN subunits altered the surface expression of YFP-hGluN1-1a/hGluN2A receptors compared with YFP-hGluN1-1a/hGluN2A receptors containing only a single mutated hGluN1 or hGluN2A subunit (Fig. 8*D*–*F*). Specifically, YFP-hGluN1-1a-S688P/hGluN2A-S511A receptor exhibited no difference in relative surface expression compared with WT YFP-hGluN1-1a/hGluN2A receptor, differing from the relative surface expression of both YFP-hGluN1-1a-S688P/hGluN2A and YFP-hGluN1-1a/hGluN2A-S511A receptors (Fig. 9*D*). YFP-hGluN1-1a-S688P/hGluN2A-S511P receptor exhibited a ∼47% reduction compared with WT YFP-hGluN1-1a/hGluN2A receptor, distinct from the values for YFP-hGluN1-1a-S688P/hGluN2A and YFP-hGluN1-1a/hGluN2A-S511P receptors (Fig. 9*E*). Finally, YFP-hGluN1-1a-S688P/hGluN2A-S511L receptor showed a ∼97% reduction in surface expression compared with WT YFP-hGluN1-1a/hGluN2A receptor, also differing from YFP-hGluN1-1a-S688P/hGluN2A and YFP-hGluN1-1a/hGluN2A-S511L receptors (Fig. 9*F*). These findings indicate that pathogenic variants and other selected mutations in the LBDs of hGluN1 and hGluN2A subunits additively or synergistically regulate the surface expression of YFP-hGluN1/hGluN2A receptors.

We next investigated whether the presence of pathogenic variants and other mutants in the LBDs of GluN1 and GluN2A subunits also affect the surface expression of GluN1/GluN2A receptors containing closed-cleft conformations induced by artificial disulfide bonds. We used rat GluN1-4a-N499C-Q686C and GFP-GluN2A-K487C-N687C constructs, which were functionally validated by electrophysiology in Figure 4. Similar to the relative surface expression patterns observed with YFP-hGluN1-1a/hGluN2A receptors carrying mutations in the LBDs, the GluN1-4a-N499C-Q686C-S688Y/GFP-GluN2A receptor showed no change, while the GluN1-4a-N499C-Q686C-S688P/GFP-GluN2A receptor exhibited a ∼35% reduction in relative surface expression compared with the GluN1-4a-N499C-Q686C/GFP-GluN2A receptor (Fig. 9*G*). Additionally, the GluN1-4a/GFP-GluN2A-K487C-N687C-S511A receptor showed a ∼79% increase, whereas the GluN1-4a/GFP-GluN2A-K487C-N687C-S511P receptor exhibited a ∼16% decrease, and the GluN1-4a/GFP-GluN2A-K487C-N687C-S511L receptor showed a ∼73% reduction in relative surface expression compared with the GluN1-4a/GFP-GluN2A-K487C-N687C receptor (Fig. 9*H*). These findings suggest that cellular mechanisms detect the presence of various mutations in the LBDs of GluN1 and GluN2A subunits containing artificial disulfide bonds.

**Figure 10.**
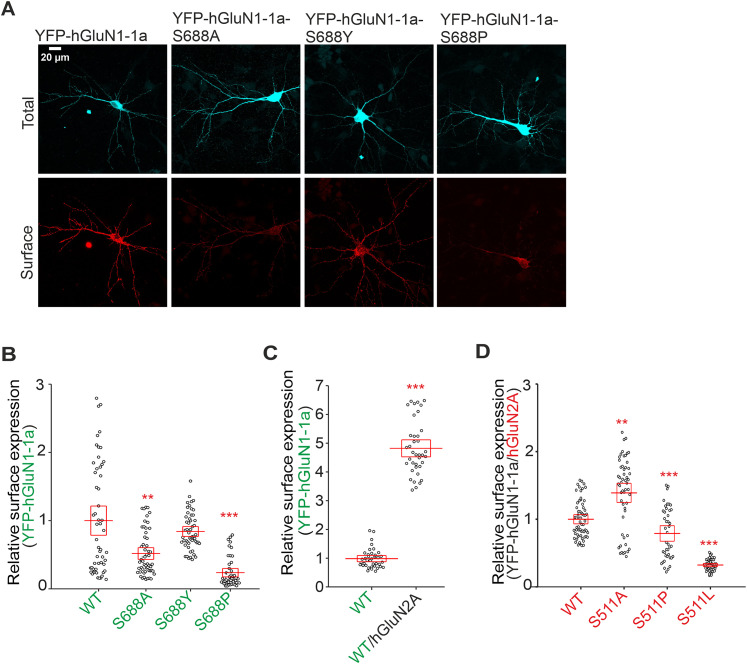
Microscopical analysis of the selected mutations of the GluN1-S688 and GluN2A-S511 residues in hippocampal neurons. ***A***, Representative images of hippocampal neurons transfected with the WT or mutated YFP-hGluN1-1a subunits. The total and the surface signal (top and bottom row, respectively) of YFP-GluN1-1a subunits were labeled using an anti-GFP antibody 48 h after the transfection. ***B***, Summary of the relative surface expression of YFP-hGluN1-1a subunits mutated at the S688 residue position, expressed in hippocampal neurons determined using fluorescence microscopy; **p < 0.010 and ***p < 0.001 versus WT; one-way ANOVA. Data points correspond to individual segments (*n* ≥ 46), and the red box plot indicates mean ± SEM. ***C***, Summary of the relative surface expression of WT YFP-hGluN1-1a alone or cotransfected with the WT hGluN2A subunit expressed in hippocampal neurons determined using fluorescence microscopy; ***p < 0.001 versus WT; Student's *t* test. Data points correspond to individual segments (*n* ≥ 38), and the red box plot indicates mean ± SEM. ***D***, Summary of the relative surface expression of WT YFP-hGluN1-1a subunit cotransfected with WT or mutated hGluN2A subunits at the S511 residue position, expressed in hippocampal neurons determined using fluorescence microscopy; **p < 0.010 and ***p < 0.001 versus WT; one-way ANOVA. Data points correspond to individual segments (*n* ≥ 42), and the red box plot indicates mean ± SEM.

Finally, we asked whether our findings on the roles that specific mutations in LBDs play in regulating the surface number of NMDARs expressed in HEK293 cells also apply to hippocampal neurons. We first determined the surface expression of YFP-hGluN1-1a subunits carrying alanine substitution and pathogenic variants of the hGluN1-S688 residue in cultured hippocampal neurons (DIV14) using an anti-GFP antibody (Fig. 10*A*). Our microscopic analysis showed the following decrease in relative surface expression of mutant YFP-hGluN1-1a subunits compared with the WT YFP-hGluN1-1a subunit: YFP-hGluN1-1a-S688A (∼48% compared with ∼23% in HEK293 cells; Fig. 10*B*) and YFP-hGluN1-1a-S688P (∼76% compared with ∼59% in HEK293 cells; Fig. 10*B*); the YFP-hGluN1-1a-S688Y subunit did not exhibit altered relative surface expression in hippocampal neurons (Fig. 10*B*). We could not directly verify the surface expression of the mutant hGluN2A subunits in hippocampal neurons because we did not have a functional tagged hGluN2A subunit. Therefore, we next transfected the WT YFP-hGluN1-1a subunit or cotransfected WT YFP-hGluN1-1a subunit with the WT hGluN2A subunit. Our microscopic analysis showed that the presence of the hGluN2A subunit profoundly increased the surface expression of the WT YFP-hGluN1-1a subunit (Fig. 10*C*). This is consistent with the previous finding that mammalian neurons produce an excess of GluN1 subunit but a limited number of GluN2 subunits (Prybylowski et al., 2002); this supports the validity of the surface expression assay with the WT YFP-hGluN1-1a subunit and untagged mutant hGluN2A subunits. Our subsequent analysis of the hippocampal neurons showed that the presence of the hGluN2A-S511A subunit led to a ∼39% increase, while coexpression with the hGluN2A-S511P and hGluN2A-S511L subunits resulted in a decrease of ∼21 and ∼68%, respectively, of the relative surface expression of the WT YFP-hGluN1-1a subunit compared with the WT hGluN2A subunit (Fig. 10*D*). These findings support the relevance of our conclusions about regulating the surface numbers of the NMDARs obtained using HEK293 cells.

## Discussion

Previous studies of early trafficking of GluN1/GluN2 receptors studied a limited number of alanine substitutions that directly interact with glycine in the GluN1 subunit or l-glutamate in the GluN2A subunit. Specifically, the GluN1-D732A/GluN2A receptor showed a ∼90% reduction in surface expression, which was associated with a ∼35,000-fold increase in the EC_50_ value for glycine measured for the GluN1-D732A/GluN2B receptor expressed in *Xenopus* oocytes (Williams et al., 1996; Kenny et al., 2009). Our experiments with the GluN1-D732A/GluN2A receptor also showed a ∼90% reduction in surface expression, although we did not detect any current responses from transfected HEK293 cells. Previously, the GluN1/GluN2B-E413A receptor showed a ∼80% reduction in surface expression (She et al., 2012) and a ∼237-fold increase in EC_50_ value for l-glutamate (Laube et al., 2004). Consistent with this, our data with the GluN1/GluN2A-E413A receptor showed a ∼80% reduction in surface expression and a ∼287-fold increase in EC_50_ value for l-glutamate. In addition, EC_50_ values were also previously calculated for the following mutants of GluN1/GluN2A receptors: GluN1-F484A/GluN2A (glycine, 3,100 μM vs our measured value of 3,624 μM; Kvist et al., 2013), GluN1-R523A/GluN2A (glycine, 5,400 μM vs our measured value of 10,261 μM; Kvist et al., 2013), GluN1/GluN2A-S511A (l-glutamate, 146 μM vs our measurement of 235 μM; Chen et al., 2005), GluN1/GluN2A-H485A (l-glutamate, 490–1,025 μM vs our measurement of 3,969 μM; Anson et al., 1998; Chen et al., 2005; Maier et al., 2007), GluN1/GluN2A-T513A (l-glutamate, 482 μM vs our measurement of 809 μM; Chen et al., 2005), GluN1/GluN2A-R518A (l-glutamate, >3,000 μM vs our measurement of 8,895 μM; Erreger et al., 2007), GluN1/GluN2A-S689A (l-glutamate, ∼2–5 μM vs our measurement of 7 μM; Anson et al., 1998; Erreger et al., 2007), and GluN1/GluN2A-T690A (l-glutamate, 2,967 μM vs our measurement of 5,254 μM; Anson et al., 1998). The observed differences in EC_50_ values can be explained by the use of different expression systems (*Xenopus* oocytes vs HEK293 cells), electrophysiological methods (two-electrode voltage clamp vs whole-cell patch clamp), and differences in the composition of the recording solutions. Our series of alanine substitutions showed a statistically significant (mutant GluN1 subunit) and no (mutant GluN2A subunit) correlation between surface expression levels of GluN1/GluN2A receptors and their EC_50_ values. Thus, our data showed that replacing critical amino acid residues within the LBDs of the GluN1 subunit with a small uncharged alanine does not, at least in the case of the GluN2A subunit, support the previously hypothesized link between surface expression levels and the agonist potency of GluN1/GluN2 receptors. In addition, we found that coexpression of both GluN1 and GluN2A subunits with alanine substitutions in LBDs exhibited an additive or synergistic effect on reducing the surface expression level of GluN1/GluN2A receptors, leading to the conclusion that LBDs of GluN1 and GluN2 subunits contribute independently to the regulation of the surface numbers of NMDARs. Our experiments with the closed-cleft conformation of LBDs (Blanke and VanDongen, 2008; Kussius and Popescu, 2010; Dai and Zhou, 2016) confirmed the additive or synergistic effect of the LBDs of the GluN1 and GluN2A subunits on the regulation of the surface numbers of the GluN1/GluN2A receptors. Our subsequent experiments revealed that closed-cleft conformation of the LBDs of GluN1 and GluN2A subunits did not mask the reducing effect of alanine and other substitutions on the surface numbers of GluN1/GluN2A receptors. We further showed that incubating the transfected HEK293 cells with a panel of competitive antagonists did not alter the surface numbers of WT GluN1/GluN2A receptors. This result is consistent with previous data on the effect of DCKA on the early trafficking of WT GluN1/GluN2A receptor (Kenny et al., 2009). Our experiments with alanine substitutions in the LBDs of GluN1/GluN2B receptors revealed similar findings to those observed with GluN1/GluN2A receptors, which suggests an evolutionarily shared mechanism for the regulation of the early trafficking of these two major diheteromeric subtypes of NMDARs.

Using the synchronized release of NMDARs from the ER, we showed that GluN1/GluN3A receptor colocalized with GA structures, similar to GluA1 receptor (Hangen et al., 2018). Consistently with a previous study showing diffuse localization of GluN1/GluN2A receptor in HEK293 cells (Hayashi et al., 2009), we did not detect GluN1/GluN2A receptors in GA, even at 15, 30, 45, and 60 min after addition of AL. Thus, the GluN1/GluN2A receptor may bypass the GA, as shown in a previous study with a transfected GluN1 subunit (Jeyifous et al., 2009). It was previously demonstrated that the WT GluN1/GluN2B receptor colocalizes with GA in COS-7 cells (She et al., 2012). Our pilot experiment did not detect WT GluN1/GluN2B receptors in GA structures using the ARIAD system; this discrepancy may be explained by the fact that expression of recombinant GluN1/GluN2B receptors may cause defragmentation of GA structures (Nakagomi et al., 2008) or cause excitotoxic damage (Zhou et al., 2013), which could affect the rate of colocalization between GA and NMDARs.

Regarding non-alanine substitutions in the GluN1 subunit, a previous study reported that the GluN1-D732E/GluN2A receptor did not show altered surface expression while exhibiting a ∼1,796-fold increase in EC_50_ value for glycine (Williams et al., 1996; Kenny et al., 2009), consistently with our results showing no difference in surface expression and ∼1,457-fold increase of EC_50_ value for glycin. In addition, our recent study reported that the pathogenic GluN1-S688Y variant did not alter the surface expression of the GluN1/GluN2A receptor but increased the EC_50_ values for glycine to 2,122 μM (Skrenkova et al., 2020), consistently with our novel data. Regarding the published data with non-alanine substitutions in the GluN2A subunit, an earlier study reported a 50% decrease, and we observed an 88% decrease in surface expression of the GluN1/GluN2A-R518H receptor (this difference may be explained by the use of colorimetric vs microscopic assays). Neither study detected current responses in HEK293 cells transfected with GluN1/GluN2A-R518H receptor (Swanger et al., 2016). In addition, the pathogenic GluN2B-E413G variant reduced surface expression of the GluN1/GluN2B receptor by ∼82% and increased the EC_50_ value for l-glutamate by ∼237-fold, which is consistent with our data with the GluN1/GluN2A-E413A receptor showing 71% lower relative surface expression and ∼288-fold higher EC_50_ value for l-glutamate. The mutant cycle of GluN1-S688 residue showed distinct levels of relative surface expression of GluN1/GluN2A receptors that correlated with EC_50_ value for glycine. Consistent with our data with alanine substitutions, the surface expression levels of GluN1/GluN2A receptors containing the mutant cycle of the GluN2A-S511 residue did not correlate with the EC_50_ values for l-glutamate. Our in silico analysis revealed a correlation between the relative surface expression values and Δ*G*_binding_ values as well as with the structural features represented by RMSD and SASA for the GluN1-S688 residue mutant cycle. We hypothesize that the structural changes of LBD of the GluN1 subunit, but not the functional properties, are the critical parameters sensed during the early trafficking of the GluN1/GluN2A receptors. This is consistent with our observations that the GluN1-D732E/GluN2A receptor was present on the cell surface similar to the WT GluN1/GluN2A receptor but had a ∼1,457-fold increased EC_50_ value for glycine. The correlation between the relative surface expression and the sensitivity of the LBD of the GluN1 subunit to glycine is in agreement with our previous study with the GluN1/GluN3A receptor (Skrenkova et al., 2019). The lack of correlation between the relative surface expression values and Δ*G*_binding_, RMSD, and SASA values observed with the GluN2A-S511 residue mutant cycle indicates the distinct mechanism underlying the regulation of the early trafficking of the GluN1/GluN2A receptors containing the mutations within the LBD of the GluN2A subunit compared with the GluN1 subunit. We also suggest that the functional properties of the GluN1/GluN2A receptors containing the mutations within the LBD of the GluN2A subunit are not critical parameters assessed during the ER processing, as (1) the GluN1/GluN2A-S511A receptor showed a ∼43% increase of surface expression but a ∼34-fold increased EC_50_ value for l-glutamate and (2) the GluN1/GluN2A-S511N receptor showed regular surface expression but did not elicit any current responses in HEK293 cells. However, combining our experimental and in silico data with >80 mutant GluN1/GluN2 receptors has not enabled us to formulate a unified principle describing how the LBDs regulate the ER release of GluN1/GluN2 receptors. We propose that future studies should include in silico studies involving the full-length NMDARs.

Previous studies identified other regions in GluN subunits besides LBDs, including ATD (Qiu et al., 2009), M3 domains (Horak et al., 2008), and CTDs (Standley et al., 2000; Scott et al., 2001; Xia et al., 2001; Horak and Wenthold, 2009), containing ER retention and export signals. We anticipate that future studies will investigate at what level other ER retention and export signals contribute to regulating the surface expression of the GluN1/GluN2 receptors with mutated LBDs. Further studies will also have to investigate the impact of the pathogenic variants in LBDs of the GluN subunits during CNS development and postnatally.
